# Genotypic resilience and fruit quality responses of tomato (*Solanum lycopersicum* L.) in progressive salinity stress across diverse cultivation conditions

**DOI:** 10.3389/fpls.2026.1786599

**Published:** 2026-06-16

**Authors:** Navdeep Singh, Dilpreet Talwar, Kulbir Singh, Babanjeet, Salesh Kumar Jindal, Jagdish Singh, Prakash Mahala, Jyoti Verma

**Affiliations:** 1 Department of Vegetable Science, Punjab Agricultural University, Ludhiana, Punjab, India; 2 Punjab Agricultural University, Regional Research Station, Gurdaspur, Punjab, India; 3 Punjab Agricultural University, Regional Research Station, Abohar, Punjab, India

**Keywords:** membrane stability, MGIDI, osmotic adjustment, root biomass, salinity stress

## Abstract

Tomato is a worldwide significant vegetable crop mainly grown for its nutritional and nutraceutical value, but its productivity is severely constrained by soil salinity. This study aimed to evaluate the performance of 20 tomato genotypes with graded salinity (0–6 dS m^−1^) in both field and pot conditions. The experiment was conducted using a split-plot design with salinity levels as main plots and genotypes as subplots, replicated thrice over two growing seasons. Genotypic variations were apparent as PTNI-203, PTNI-202, and PTNI-8 exhibited better vegetative development, earlier flowering, larger fruit, and dense pericarp thickness under high salinity. Root fresh and dry weights were maximized in PTNI-11, PTNI-25, and PTNI-20, indicating good osmotic adjustment and salt-tolerant processes. Physiological and biochemical profiling further revealed substantial genotypic variation in osmolyte accumulation, pigment stability, and membrane integrity under saline conditions. These genotypes also recorded 30%–40% higher proline, 15%–20% higher protein content, and membrane stability indices above 85. The total yield was reduced gradually with the salinity, and the tolerant genotypes (PTNI-203, PTNI-8, and PTNI-202) maintained higher yield (524–540 q acre^−1^), representing 55%–65% higher productivity than susceptible genotypes under 6 dS m^−1^. Rank analysis supported the fact that genotype was the major factor that determined growth and productivity traits with salinity imposing secondary inhibitory effects. This study provides practical insights into genotype-specific adaptation to salinity stress and identifies promising tomato lines for sustaining productivity and contributing to climate-resilient horticultural practices. The identified genotypes can serve as promising candidates for cultivation and breeding programs in salt-affected regions.

## Introduction

Tomato (*Solanum lycopersicum* L.) is the second most produced vegetable crop in the world after potato (Collins et al., 2022). Tomato was domesticated in Central America and has been valued because of its nutritional and nutraceutical values providing vitamins (A, C, and folate), minerals (K and Mn), and bioactive compounds (lycopene and β-carotene) that are essential to human wellbeing and fruit coloration (Mallick et al., 2021; Peralta and Spooner, 2000; Wang et al., 2023).

Abiotic stresses, especially soil salinity, are harmful to tomato productivity. Salinity impairs water absorption and ionic homeostasis, thus causing osmotic and ionic stresses that limit plant development, photosynthesis, and yield (Roșca et al., 2023). Salinity affects approximately 20% of the cultivated land globally (Hu and Schmidhalter, 2005; Shrivastava and Kumar, 2015). Moderately salt-tolerant tomato has a salinity threshold of approximately 1.7 dS m^−1^ and can partially stabilize osmotic balance by selectively absorbing K^+^ and limiting Na^+^ accumulation, but long-term or high salinity has adverse effects on germination (Maas and Hoffman, 1977).

It has been reported since early studies that moderate salinity can increase specific nutritional characteristics in tomato fruits (Mizrahi, 1982; Cuartero and Fernández-Muñoz, 1998). Osmotic adaptation by salt and antioxidant defense mechanism activation have been reported to boost lycopene, carotenoids, and phenolic compounds, but these outcomes are extremely genotype-dependent (Carrasco-Gil et al., 2016; Kiferle et al., 2022). On the other hand, high salinity lowers the size of fruits, yield components, and mineral levels (K, P, Mg, and Zn) because of the ionic competition and thus points out the trade-off between nutritional benefit and productivity (Toscano et al., 2019).

The salinity response of tomato depends on genotype × environment interaction. Salt-tolerant genotypes are often characterized by the limited translocation of Na^+^ and Cl^−^ to shoots, delayed leaf senescence, hormonal balance, and constant vegetative and reproductive performance in response to salt (Juan et al., 2005; Roșca et al., 2023). This kind of physiological strength is required to sustain leaf area, pericarp thickness, fruit morphology, root biomass, yield components, and marketable yield in a saline environment. In addition, field/pot cultivation differences can affect plant growth, canopy structure, root development, and yield distribution (Atta et al., 2023; Fikre and Boto, 2024).

With the rising level of soil salinity and its negative effects on tomato production, there is an urgent need to find genotypes that are region-specific and salt-tolerant (Ali et al., 2021; Alatawi et al., 2025). Therefore, a clear research gap exists in identifying tomato genotypes that exhibit stable, multi-trait salinity tolerance under realistic and variable growing environments. In particular, there is limited information on how genotype × environment interactions influence the integration of growth, root traits, biochemical responses, and yield stability under graded salinity stress. We hypothesize that tomato genotypes differ significantly in their ability to tolerate salinity stress and that salt-tolerant genotypes maintain higher growth, root biomass, biochemical stability, and yield through coordinated physiological and antioxidant mechanisms under increasing salinity levels. Accordingly, the objectives of this study were to evaluate tomato genotypes under graded salinity levels (0–6 dS m^−1^) in both field and pot conditions and to identify superior genotypes based on integrated morpho-physiological, biochemical, and yield traits.

## Materials and methods

### Experimental site and design

The study was conducted at the Vegetable Research Farm of the Department of Vegetable Science at Punjab Agricultural University (PAU), Ludhiana, India (30.90° N latitude, 75.85° E longitude, altitude 247 m above mean sea level) during the 2021–2022 and 2022–2023 crop seasons. During the experimental period, temperature ranged from 18 to 35 °C and relative humidity ranged from 50% to 85%. Twenty tomato genotypes were selected from an initial pool of 155 genotypes based on germination percentage, seedling survival rate, and phenotypic performance under preliminary screening conditions. All genotypes used in the study were determinate in growth habit and were obtained from PAU, Ludhiana, India except for two genotypes (Roma and Edkawi), which were sourced from the United States of America.

Seedlings were raised in plug trays (98-cell) filled with a sterilized growing medium under controlled nursery conditions with a maintained temperature of 25 ± 2 °C and a relative humidity of 70%–80%. Seedlings were transplanted at 25–30 days after sowing at four to five true leaf stages. For the pot experiment, plants were grown in plastic grow bags (25 × 25 × 25 cm; upper diameter × lower diameter × height). Each grow bag was filled with approximately 8.6 kg of growing medium. The growing medium consisted of soil, sugarcane press mud, and well-decomposed farmyard manure (FYM) mixed in a 1:1:1 ratio (w/w) for balanced medium for nutrient supply, aeration, and water-holding capacity. Pots were maintained under open conditions with natural light. Field-grown seedlings were transplanted at a spacing of 75 × 30 cm (row × plant) following the PAU package of practices. The field was prepared through ploughing and leveling and well-decomposed FYM (10 t acre^-1^) along with recommended doses of fertilizers (N:P_2_O_5_:K_2_O @ 60:25:25 kg acre^-1^) were applied. Irrigation, staking, pruning, weeding, and plant protection measures were carried out uniformly as per PAU recommendations.

Salt stress experiments were conducted in both pot and field settings using the saline irrigation water that had been prepared using analytical grade salts with a ratio of 2:1:1 of sodium chloride (NaCl), calcium chloride (CaCl_2_, anhydrous), and magnesium sulfate heptahydrate (MgSO_4_.7H_2_O) on weight basis (w/w). The salts were gradually added to irrigation water, and electrical conductivity (EC) was monitored using a conductivity meter until the desired salinity levels were achieved. The final EC levels corresponded to the different salinity treatments.

Preliminary calibration experiments were performed to establish the relationship between salt addition and EC of irrigation water. Based on calibration, approximately 1.05 g L^−1^ of salt mixture increased EC to approximately 2 ds m^−1^ and higher salinity levels were achieved by proportionally increasing the salt concentration. The corresponding molar concentrations of individual salts for each salinity treatment are presented in **Table 1**. For field conditions, preliminary measurements were conducted to determine the irrigation volume required to reach field capacity for each plot, which was approximately 42 L plot^−1^. Soil EC was periodically monitored before irrigation, and if a decline in EC was observed due to leaching or rainfall, the required amount of salt mixture was reapplied to irrigation water to maintain the target salinity for each treatment. The calculated amount of salt mixture corresponding to each salinity treatment was dissolved in this volume of irrigation water and applied uniformly to maintain salinity levels. The experiment was conducted in triplicate in a split plot design with salinity level as the main plot and genotypes as subplots. Salinity regimes used included a (T_1_) non-saline control, (T_2_) 2 dS m^−1^, (T_3_) 4 dS m^−1^, and (T_4_) 6 dS m^−1^.

**Table 1:** Composition of saline irrigation solutions used to establish different salinity treatments in tomato.

Salinity level	Salt mixture (g L^-1^)	NaCl (mM)	CaCl2 (mM)	MgSO4.7H2O (mM)
Control	0.00	0.00	0.00	0.00
2 (dS m^-1^)	1.05	8.98	2.37	1.06
4 (dS m^-1^)	2.10	17.96	4.74	2.12
6 (dS m^-1^)	3.15	26.94	7.11	3.18

Saline solutions were prepared using NaCl, CaCl_2_ (anhydrous), and MgSO_4_.7H_2_O in a 2:1:1 ratio (w/w). Salt concentrations were determined through preliminary calibration relating salt addition to electrical conductivity (EC).

### Growth and vegetative traits

The vegetative growth indices were recorded 30, 60, and 90 days after transplantation (DAT). The plant height was determined as the distance between the base and apex of the terminal leaf, and the count of the leaf in a plant was determined at the time of harvest. Proxies to the photosynthetic surface area and overall vegetative architecture were determined as leaf area. Canopy area was calculated as π × (canopy width/2)^2^. Phenological information included the time taken to first flower, which was the time period between transplanting and the first flower completely opening.

### Fruit and root morphology

Fruits were harvested at the red-ripe stage based on uniform color and firmness. Fruit morphological features were recorded in five random fruits of each treatment during harvest such as polar and equatorial diameter and pericarp thickness using a Digital Vernier calliper (Mitutoyo, Japan; accuracy ±0.01 mm). Root attributes were assessed by measuring fresh root weight as is at the end of harvest after which samples were dried at 65 ± 2°C to determine dry root biomass, hence providing information on belowground growth processes and salt stress responses.

### Yield assessment

The total and marketable yield were calculated and weights of all the fruits collected were averaged across the replicates and divided by the total plant, to obtain the total and marketable fruit yield. Per-acre value was extrapolated based on the spacing of the plant and the normative field conversion factors. Marketable yield covered the fruits that met the set standards of size, shape, and quality, thus allowing the determination of both productivity and commercial viability in saline conditions.

### Quality attributes

Ascorbic content (mg/100 mL juice) was determined using metaphosphoric acid and indophenol dye titration as described by the AOAC method. Titratable acidity (TA) (g anhydrous citric acid/100 mL juice) was measured as anhydrous citric acid using 0.1 N NaOH with a phenolphthalein indicator (Srivastava and Sit, 2024). Total soluble solids (°Brix) were measured using a hand refractometer. Chlorophyll (mg/g FW) was total carotenoids (mg/g FW) extracted with 80% acetone and absorbance measured at 665, 645, and 480 nm (Hossain and Jayadeep, 2018). Total soluble sugars (% DW) were estimated using the anthrone method and reducing sugars using the 3,5-dinitrosalicyclic acid (DNS) method (Rana et al., 2025). Total protein content (%FW) was determined using Folin reagent (Waterborg and Matthews, 1994).

Membrane stability index (%) (MSI) was assessed following the method of Sairam and Srivastava (2002) by measuring electrolyte leakage from leaf discs before and after heat treatment in distilled water using a conductivity meter. Hydrogen peroxide (H_2_O_2_) (µ/mg) was estimated spectrophotometrically following the procedure of Ozden et al. (2009) by reacting the extract with 1 M potassium iodide in phosphate buffer (pH 7.0) and measuring absorbance at 390 nm. Proline content (µmol/g DW) was quantified following the acid ninhydrin method described by Ábrahám et al. (2010). Total phenolic content (mg/100 g) (TPC) was determined using Folin–Ciocalteu reagent and absorbance was measured at 760 nm with gallic acid as standard (Tapia-Salazar et al., 2019). Lycopene (mg/g FW) was extracted with acetone:hexane (1:1, v/v) and quantified spectrophotometrically at 505 nm following Srivastava and Srivastava (2015).

### Statistical analysis

Two-way analysis of variance (ANOVA) was performed with the salinity level as the main plot and genotype as the subplot with across-year analysis using cultivation condition (field and pot in combination with year). Genotypic, environmental, and temporal effects were analyzed using the rank analysis (*R*_A_, *R*_B_, *R*_C_, and *R*_D_). Statistically significant differences between the various treatment effects were observed by separating means with the Duncan Multiple Range Test (DMRT) with a significance level at 0.05. All statistical calculations were conducted using R software with the use of the package “*agricolae*” for multiple comparison tools.

## Results

### Vegetative growth and plant architecture

**Figure 1:**
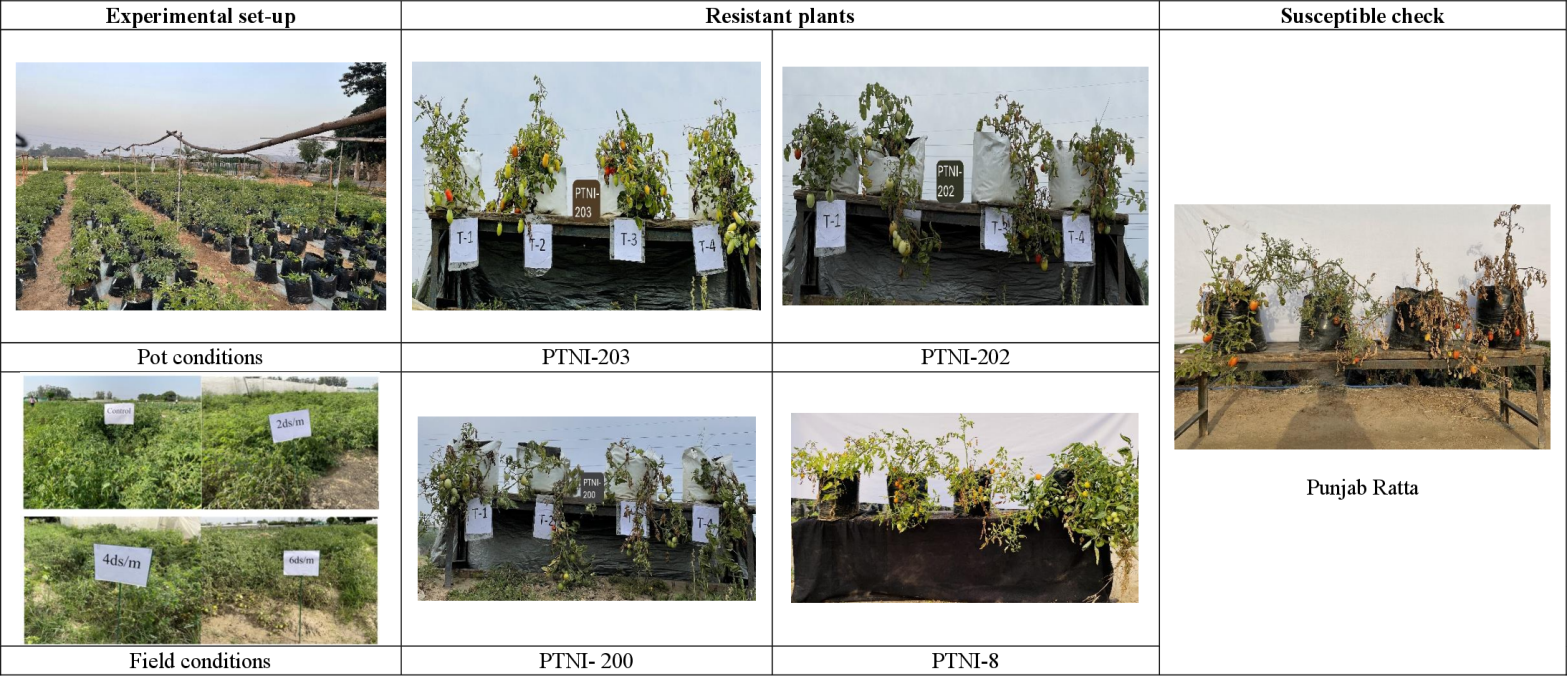
Experimental setup showing pot and field conditions used to evaluate tomato genotypes under graded salinity stress and representative phenotypic differences between tolerant and susceptible lines.

Genotype and stress interaction led to the dynamic development of tomato in salty conditions, as shown in **Figure 1**. The early vegetative growth was largely limited by salinity with the plant height reducing by 29.2 to 18.2 cm, representing a decline of approximately 38% under control and salinity conditions, respectively. The impact of environmental and seasonal factors was insignificant (*R*_C_ =1.74; *R*_D_ = 0.33). The genotypic potential was the key factor in the growth of plants as they produced bigger (*R*_A_ = 25.97 at 60 DAT and *R*_A_ = 34.71 at 90 DAT), with tolerant entries able to maintain higher growth rates despite high salinity stress (*R*_B_ = 23.10 and *R*_B_ = 22.57) and sensitive genotypes were weakened (**Table 2**).

**Table 2:** Effects of genotypes, salinity levels, environment, and year on tomato growth parameters based on Taguchi orthogonal array analysis.

Factor	Level	Values	Plant height (cm) 30 DAT	Plant height (cm) 60 DAT	Plant height (cm) 90 DAT	Leaf area (cm2)	Canopy width (cm2)	Days to 50% flowering	Pericarp thickness (mm)
A (Genotypes)	PTNI-27	KA1	24.13 ± 1.14^b^	65.53 ± 2.56^b^	88.73 ± 2.55^bc^	7.48 ± 0.24^g^	39.39 ± 1.54^c^	49.69 ± 1.76^b^	5.59 ± 0.11^c^
	PTNI-59	KA2	23.56 ± 1.33^bc^	65.02 ± 2.58^b^	88.09 ± 2.50^c^	7.20 ± 0.23^b^	38.99 ± 1.68^c^	49.56 ± 1.65^b^	5.56 ± 0.11^c^
	PTNI-8	KA3	25.88 ± 1.15^a^	68.43 ± 2.31^ab^	92.98 ± 2.26^ab^	7.46 ± 0.26^f^	41.94 ± 1.50^b^	47.38 ± 1.01^c^	6.11 ± 0.13^ab^
	PTNI-54	KA4	25.01 ± 1.15^ab^	67.54 ± 2.51^ab^	90.98 ± 2.39^ab^	7.86 ± 0.20^I^	40.43 ± 1.41^b^	47.50 ± 1.36^c^	5.90 ± 0.13^b^
	PTNI-203	KA5	26.64 ± 1.11^a^	70.40 ± 2.27^a^	95.11 ± 2.27^a^	7.66 ± 0.24^h^	43.19 ± 1.25^a^	46.69 ± 1.34^c^	6.25 ± 0.13^a^
	PTNI-67	KA6	25.64 ± 1.10^a^	67.59 ± 2.34^ab^	91.78 ± 2.24^ab^	7.96 ± 0.18^j^	41.80 ± 1.15^b^	48.13 ± 1.47^bc^	5.97 ± 0.14^b^
	PTNI-3	KA7	25.33 ± 1.19^a^	67.72 ± 2.44^ab^	91.29 ± 2.31^ab^	7.28 ± 0.24^c^	40.78 ± 1.42^b^	47.94 ± 1.42^bc^	5.94 ± 0.13^b^
	PTNI-202	KA8	25.93 ± 1.12^a^	69.09 ± 2.21^a^	93.51 ± 2.25^ab^	8.11 ± 0.19^k^	43.17 ± 1.37^a^	46.81 ± 1.19^c^	6.16 ± 0.14^ab^
	PTNI-2	KA9	24.96 ± 1.05^ab^	66.89 ± 2.41^b^	90.19 ± 2.39^ab^	7.18 ± 0.20^a^	40.26 ± 1.42^b^	48.50 ± 1.59^bc^	5.83 ± 0.12^b^
	PTNI-200	KA10	25.56 ± 1.10^a^	68.26 ± 2.36^ab^	92.31 ± 2.22^ab^	7.90 ± 0.20^l^	42.68 ± 0.92^ab^	47.25 ± 1.18^c^	6.02 ± 0.13^ab^
	PTNI-11	KA11	24.24 ± 1.17^b^	65.89 ± 2.48^b^	89.08 ± 2.51^bc^	7.68 ± 0.24^h^	39.28 ± 1.48^c^	48.31 ± 1.43^bc^	5.59 ± 0.11^c^
	PTNI-25	KA12	23.43 ± 1.29^bc^	62.29 ± 2.50^bc^	85.14 ± 2.65^c^	7.28 ± 0.25^c^	38.09 ± 1.55^c^	49.56 ± 1.63^b^	5.44 ± 0.11^c^
	PTNI-22	KA13	24.27 ± 1.08^b^	66.14 ± 2.48^b^	89.31 ± 2.49^bc^	7.33 ± 0.25^d^	39.41 ± 1.41^c^	48.75 ± 1.59^bc^	5.68 ± 0.11^b^
	Roma	KA14	24.92 ± 1.10^ab^	66.73 ± 2.42^b^	89.85 ± 2.47^bc^	7.21 ± 0.22^b^	40.28 ± 1.49^b^	48.13 ± 1.55^bc^	5.75 ± 0.11^b^
	PTNI-20	KA15	23.61 ± 1.36^bc^	62.84 ± 2.47^bc^	85.92 ± 2.51^c^	7.75 ± 0.24^m^	38.59 ± 1.58^c^	49.44 ± 1.63^b^	5.50 ± 0.12^c^
	PTNI-19	KA16	23.32 ± 1.41^bc^	64.18 ± 2.54^bc^	87.33 ± 2.50^c^	7.07 ± 0.22^ab^	39.29 ± 1.62^c^	49.69 ± 1.65^b^	5.52 ± 0.11^c^
	Edkawi	KA17	24.53 ± 1.00^b^	66.59 ± 2.46^b^	89.19 ± 2.42^bc^	7.09 ± 0.21^ab^	40.01 ± 1.55^b^	47.94 ± 1.46^bc^	5.71 ± 0.11^b^
	PTNI-69	KA18	23.31 ± 1.29^bc^	63.63 ± 2.55^bc^	86.88 ± 2.54^c^	6.83 ± 0.22^n^	38.64 ± 1.52^c^	49.31 ± 1.57^b^	5.50 ± 0.12^c^
	Punjab Chhuhara	KA19	18.54 ± 2.16^d^	52.90 ± 5.07^d^	73.49 ± 5.61^d^	6.02 ± 0.19^o^	33.74 ± 2.47^d^	50.00 ± 1.25^a^	4.66 ± 0.29^d^
	Punjab Ratta	KA20	17.17 ± 1.81^e^	44.43 ± 4.60^e^	60.40 ± 6.18^e^	4.22 ± 0.18^p^	28.62 ± 2.93^e^	52.75 ± 1.53^a^	3.83 ± 0.38^e^
		RA	9.47	25.97	34.71	3.89	14.57	6.06	2.43
B (Salt concentration)	(T1) Control	KB1	29.20 ± 0.46^ab^	73.51 ± 1.51^a^	96.22 ± 0.30^a^	8.10 ± 0.09 ^a^	45.95 ± 0.65^a^	41.15 ± 0.70^d^	6.26 ± 0.04 ^a^
	(T2) 2 ds m−1	KB2	26.97 ± 0.59^ab^	69.94 ± 0.94^a^	93.42 ± 0.13^a^	7.73 ± 0.08 ^b^	43.56 ± 0.95^a^	46.55 ± 0.34^c^	5.95 ± 0.02 ^b^
	(T3) 4 ds m−1	KB3	21.65 ± 1.16^b^	64.55 ± 0.20^b^	87.01 ± 0.72^b^	6.96 ± 0.00 ^c^	35.84 ± 0.76^b^	52.49 ± 1.33^b^	5.32 ± 0.12 ^c^
	(T4) 6 ds m−1	KB4	18.18 ± 1.82^c^	50.41 ± 5.24^c^	73.65 ± 6.09^c^	6.12 ± 0.02 ^d^	32.37 ± 1.67^c^	54.48 ± 1.35^a^	4.97 ± 0.13 ^d^
		RB	11.02	23.10	22.57	1.98	13.58	13.33	1.29
C (Environment)	Field conditions	KC1	24.87 ± 1.01a	65.75 ± 1.6a	90.67 ± 1.81a	7.23 ± 0.28a	40.05 ± 1.52a	47.63 ± 1.70b	5.75 ± 0.17a
	Pot conditions	KC2	23.13 ± 2.29a	63.46 ± 5.06a	84.48 ± 4.87b	7.22 ± 0.30a	38.81 ± 2.69a	49.71 ± 2.35a	5.50 ± 0.22b
		RC	1.74	2.29	6.19	0.01	1.24	2.08	0.25
D (Year)	2021–2022	KD1	23.83 ± 1.78a	64.44 ± 3.76a	87.52 ± 3.78a	7.22 ± 0.30a	39.22 ± 2.01a	48.29 ± 1.90 a	5.56 ± 0.22a
	2022–2023	KD2	24.16 ± 1.82a	64.76 ± 3.81a	87.63 ± 3.93 a	7.23 ± 0.29a	39.64 ± 2.38 a	49.04 ± 2.25 a	5.69 ± 0.18a
		RD	0.33	0.32	0.11	0.01	0.42	0.75	0.13
Rank			B>A>C>D	A>B>C>D	A>B>C>D	A>B>C≥D	A>B>C>D	B>A>C>D	A>B>C>D
Major contributing factor			Factor B	Factor A	Factor A	Factor A	Factor A	Factor B	Factor A

Mean value ± standard error of three replicates, mean values within a column with different letters (a–d) are significantly different at *p* ≤ 0.05. Factors A, B, C, and D represent the effect of genotypes, salt concentration, environment, and year, respectively. *R*_A_, *R*_B_, *R*_C_, and *R*_D_ were the differences between the highest and lowest values of *K_ij_* (average value of the respective parameter, *i* = A, B, C, and D and *j* = 1, 2, …9) of each parameter.

Canopy width was primarily affected by genotype (*R*_A_), with PTNI-203 and PTNI-202 exhibiting broad canopies (approximately 43 cm^2^) and sensitive genotypes being confined (approximately 28–34 cm^2^). The secondary inhibitory effect was influenced by salinity (*R*_B_), and there was no significant effect of the environmental and seasonal factors. The phenotype in genotype-dominated resilience to saline environments is highlighted in this hierarchy (A>B>C>D). The same pattern was observed with leaf area with the first effect mainly being genotype-dependent (*R*_A_ = 3.89) and the second factor of decrease being salinity (*R*_B_ = 1.98). Genotypes that were tolerant such as PTNI-20, PTNI-67, and PTNI-202 retained greater leaf areas (7.8–8.1 cm^2^) compared to the susceptible checks, e.g., Punjab Ratta and Punjab Chhuhara, which were reduced (4.2–6.0 cm^2^). High stress (*R*_B_) increased flowering by 41.2–54.5 days (32% increase) and genotype affected inherent earliness (*R*_A_), with tolerant genotypes like PTNI-203, PTNI-202, and PTNI-8 showing flowering earlier than sensitive genotypes.

### Morphology of fruits and pericarp thickness

**Figure 2:**
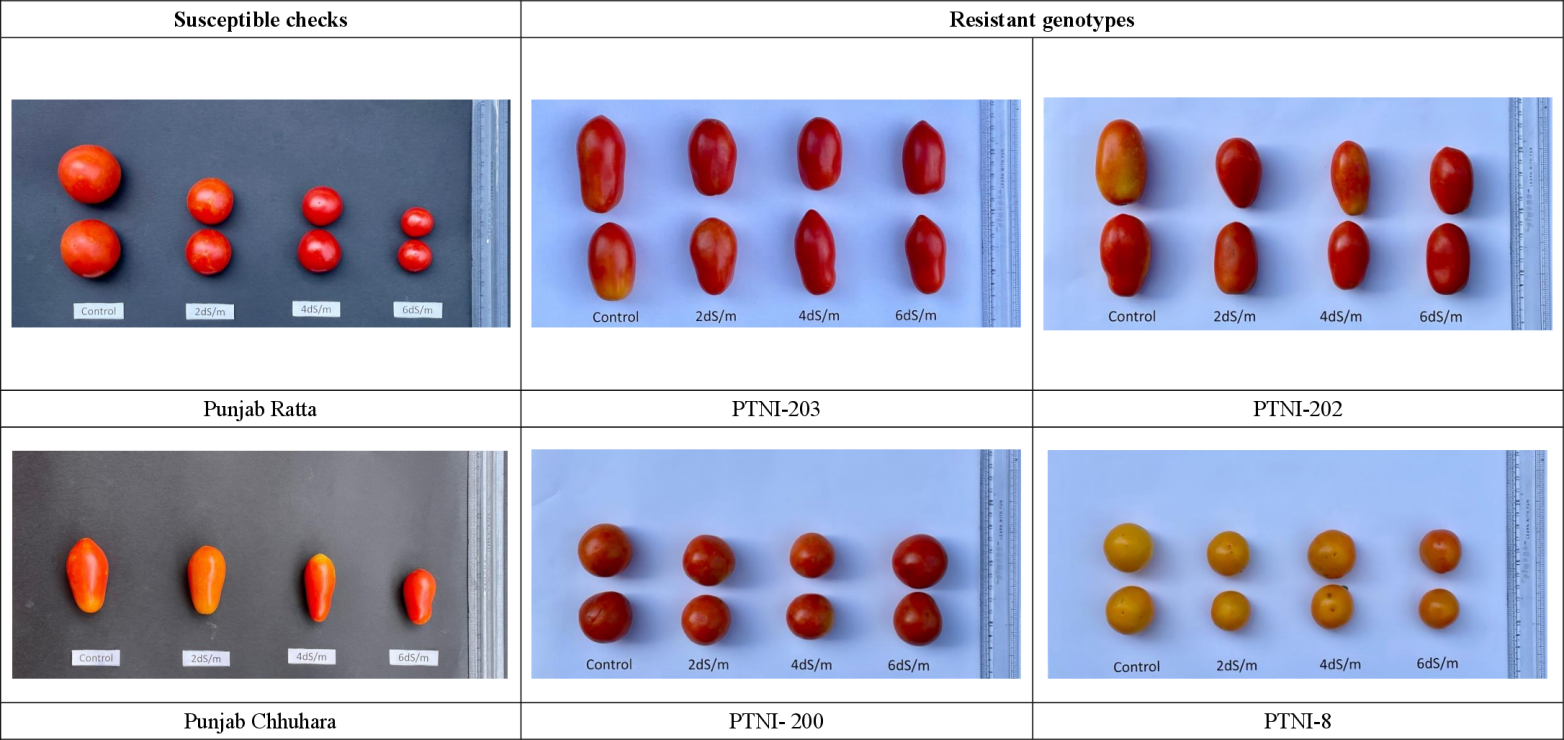
Comparative fruit morphology of susceptible (Punjab Ratta and Punjab Chhuhara) and salt-tolerant tomato genotypes (PTNI-203, PTNI-202, PTNI-200, and PTNI-8) across different salinity levels (0–6 dS m^-1^).

Fruit morphology was predominantly influenced by genotype, with polar diameter (RA = 2.51) and equatorial diameter (RA = 3.17) which showed greater genotypic variation, while salinity exerted a secondary inhibitory effect on these traits (RB = 1.06 and 1.13, respectively), as illustrated in **Figure 2**. Tolerant genotypes like PTNI-20, PTNI-25, and PTNI-3 continued to have long and wide fruits (polar 5.5–6.2 cm; equatorial 5.0–6.2 cm) and the rest of all other entries experienced salt stress. The same trend was also observed in the pericarp thickness, which was mainly genotype-controlled (*R*_A_ = 2.43) and thinned by salinity (*R*_B_ = 1.29) where PTNI-203, PTNI-8, and PTNI-200 had higher pericarp thickness (average of 5.0–6.3 mm) and the susceptible genotypes had reduced fruit dimensions (average of 3.8–4.7 mm) (**Table 2**).

### Reproductive performance and yield attributes

Genotype has a stronger influence on the number of fruits per plant (NFPP) (*R*_A_ = 12.31), then salinity (*R*_B_ = 4.80) with minor roles played by cultivation and season. The genotypes, including PTNI-27, PTNI-22, and PTNI-8, were found to be of high performance with each genotype bearing over 34 fruits on the plant, whereas genotypes like PTNI-19 and PTNI-203 showed 22–24 fruits. Salinity suppressed fruit set from approximately 31 in control to approximately 27 at 6 dS m^−1^ (13% reduction), which was an indicator of suppressed flower retention and reproductive physiology as depicted in **Table 3**.

**Table 3:** Relative contribution of genotypes, salinity levels, environment, and year to fruit development, root growth, and yield traits in tomato using Taguchi orthogonal design.

Factor	Level	Values	Number of fruits plant-1	Polar diameter (cm)	Equatorial diameter (cm)	Fresh weight of roots (g)	Dry weight roots (g)	Total yield (q acre−1)	Marketable yield (q acre−1)
A (Genotypes)	PTNI-27	KA1	34.58 ± 0.68^a^	3.96 ± 0.15^c^	3.36 ± 0.16^a^	65.35 ± 3.29^b^	28.91 ± 1.97^b^	457.63 ± 18.81^c^	404.69 ± 17.34^cd^
	PTNI-59	KA2	30.76 ± 0.65^bc^	3.76 ± 0.10^a^	3.62 ± 0.07^b^	63.62 ± 4.80^b^	26.21 ± 2.00^b^	444.13 ± 19.90^cd^	398.25 ± 17.94^cd^
	PTNI-8	KA3	34.66 ± 0.48^a^	4.37 ± 0.15^f^	4.12 ± 0.04^d^	60.46 ± 4.83^b^	29.47 ± 2.99^b^	523.69 ± 15.56^ab^	459.38 ± 14.07^ab^
	PTNI-54	KA4	29.99 ± 0.56^bc^	4.14 ± 0.10^e^	4.03 ± 0.14^c^	63.29 ± 4.66^b^	32.11 ± 2.46^ab^	486.44 ± 17.20^b^	433.19 ± 15.57^bc^
	PTNI-203	KA5	23.82 ± 0.52^d^	5.94 ± 0.08^m^	4.49 ± 0.11^e^	63.37 ± 4.01^b^	25.75 ± 1.84^b^	539.69 ± 16.65^a^	484.63 ± 14.03^a^
	PTNI-67	KA6	30.36 ± 0.53^bc^	4.39 ± 0.08^fg^	3.81 ± 0.08^bc^	57.40 ± 3.68^bc^	26.76 ± 1.32^b^	501.75 ± 17.24^b^	459.88 ± 15.79^ab^
	PTNI-3	KA7	25.79 ± 0.55^c^	5.55 ± 0.07^k^	5.05 ± 0.12^I^	60.54 ± 3.61^b^	24.07 ± 1.68^b^	495.88 ± 17.58^b^	444.13 ± 14.50^b^
	PTNI-202	KA8	26.11 ± 0.46^c^	5.26 ± 0.07^j^	5.04 ± 0.13^I^	62.19 ± 5.86^b^	31.42 ± 2.20^ab^	528.69 ± 16.04^ab^	463.63 ± 14.21^ab^
	PTNI-2	KA9	33.14 ± 0.55^ab^	4.21 ± 0.11^ef^	3.71 ± 0.06^b^	60.95 ± 4.94^b^	30.58 ± 2.38^ab^	481.44 ± 17.92^b^	433.06 ± 16.84^bc^
	PTNI-200	KA10	26.48 ± 0.46^c^	4.31 ± 0.06^f^	4.64 ± 0.12^f^	70.40 ± 3.25^ab^	28.72 ± 3.39^b^	511.75 ± 15.43^b^	454.06 ± 14.40^b^
	PTNI-11	KA11	30.18 ± 0.58^bc^	4.31 ± 0.11^f^	3.95 ± 0.10^c^	72.39 ± 4.75^a^	26.66 ± 1.04^b^	459.00 ± 18.70^c^	411.44 ± 17.87^c^
	PTNI-25	KA12	25.88 ± 0.68^c^	5.53 ± 0.14^k^	6.19 ± 0.19^j^	73.58 ± 4.98^a^	34.07 ± 3.42^a^	416.31 ± 17.62^d^	371.63 ± 17.58^de^
	PTNI-22	KA13	34.75 ± 0.56^a^	4.33 ± 0.11^f^	3.02 ± 0.11^a^	62.50 ± 4.71^b^	30.22 ± 1.46^ab^	466.81 ± 18.26^c^	414.00 ± 17.19^c^
	Roma	KA14	27.25 ± 0.55^c^	5.23 ± 0.15^j^	4.37 ± 0.04^e^	66.87 ± 4.55^ab^	26.49 ± 1.44^b^	476.25 ± 17.56^bc^	431.50 ± 17.13^bc^
	PTNI-20	KA15	25.46 ± 0.56^c^	6.18 ± 0.22^n^	5.55 ± 0.20^k^	68.88 ± 4.82^ab^	27.27 ± 2.07^b^	420.94 ± 17.58^d^	378.19 ± 18.00^de^
	PTNI-19	KA16	22.44 ± 0.60^e^	5.18 ± 0.11^j^	4.96 ± 0.17^h^	56.62 ± 4.95^bc^	24.83 ± 1.52^b^	434.44 ± 18.79^cd^	390.19 ± 17.10^d^
	Edkawi	KA17	29.26 ± 0.59^bc^	4.22 ± 0.09^ef^	3.83 ± 0.09^bc^	62.57 ± 4.79^b^	28.41 ± 2.03^b^	469.75 ± 18.63^bc^	420.56 ± 16.22^c^
	PTNI-69	KA18	28.86 ± 0.63^bc^	3.90 ± 0.10^b^	4.07 ± 0.15^c^	63.87 ± 5.15^b^	31.06 ± 2.51^ab^	432.69 ± 19.29^d^	382.00 ± 17.50^d^
	Punjab Chhuhara	KA19	31.20 ± 0.51^b^	4.64 ± 0.16^h^	3.57 ± 0.08^ab^	68.64 ± 3.13^ab^	37.57 ± 3.33^a^	457.13 ± 24.50^c^	395.31 ± 21.47^d^
	Punjab Ratta	KA20	28.96 ± 0.37^bc^	3.67 ± 0.34^a^	3.24 ± 0.32^a^	41.43 ± 2.53^c^	21.16 ± 1.52^c^	358.19 ± 31.88^e^	275.44 ± 28.55^f^
		RA	12.31	2.51	3.17	32.15	16.41	181.50	209.19
B (Salt concentration)	(T1) Control	KB1	31.36 ± 0.23 ^a^	5.12 ± 0.08 ^a^	4.73 ± 0.02 ^a^	72.49 ± 2.39^a^	34.25 ± 0.94^a^	554.05 ± 3.83^a^	500.61 ± 7.65^a^
	(T2) 2 ds m−1	KB2	30.72 ± 0.16 ^ab^	4.91 ± 0.07 ^b^	4.54 ± 0.01 ^b^	65.10 ± 6.75^b^	23.24 ± 2.97^c^	515.90 ± 2.55^a^	442.45 ± 3.93^b^
	(T3) 4 ds m−1	KB3	27.34 ± 0.19 ^c^	4.52 ± 0.06 ^c^	4.06 ± 0.03 ^c^	61.13 ± 8.33^c^	27.35 ± 0.84^b^	434.56 ± 2.79^b^	390.40 ± 12.12^c^
	(T4) 6 ds m−1	KB4	26.56 ± 0.18 ^c^	4.06 ± 0.04 ^d^	3.60 ± 0.01 ^d^	54.26 ± 9.76^d^	29.52 ± 0.18^b^	368.00 ± 2.44^c^	327.56 ± 2.16^d^
		RB	4.80	1.06	1.13	18.23	11.01	186.05	173.05
C (Environment)	Field conditions	KC1	29.31 ± 0.79a	4.54 ± 0.14b	4.25 ± 0.17a	74.75 ± 0.81a	30.75 ± 1.09a	473.08 ± 27.54a	412.94 ± 24.64a
	Pot conditions	KC2	28.68 ± 0.78a	4.77 ± 0.16a	4.21 ± 0.16a	51.74 ± 4.35b	26.43 ± 2.12b	463.18 ± 27.00a	417.57 ± 24.60a
		RC	0.63	0.23	0.04	23.01	4.32	9.90	4.63
D (Year)	2021–2022	KD1	29.07 ± 0.80a	4.67 ± 0.17a	4.22 ± 0.16a	64.02 ± 5.02a	28.28 ± 1.89a	468.71 ± 27.27a	423.09 ± 24.86a
	2022–2023	KD2	28.93 ± 0.79a	4.64 ± 0.15a	4.24 ± 0.17a	62.47 ± 5.65a	28.90 ± 1.85a	467.55 ± 27.40a	407.43 ± 24.05a
		RD	0.14	0.03	0.02	1.55	0.62	1.16	15.66
Rank			A>B>C>D	A>B>C>D	A>B>C>D	A>B>C>D	A>B>C>D	B>A>C>D	A>B>C>D
Major contributing factor			Factor A	Factor A	Factor A	Factor A	Factor A	Factor B	Factor A

Mean value ± standard error of three replicates, mean values within a column with different letters (a–d) are significantly different at *p* ≤ 0.05. Factors A, B, C, and D represent the effect of genotypes, salt concentration, environment, and year, respectively. *R*_A_, *R*_B_, *R*_C_, and *R*_D_ were the differences between the highest and lowest values of *K_ij_* (average value of the respective parameter, *i* = A, B, C, and D and *j* = 1, 2, …9) of each parameter.

Both genotype (*R*_A_=181.50) and salinity (*R*_B_ = 186.05) exhibited sensitivity to total yield, and salinity was more influential. PTNI-203, PTNI-8, and PTNI-202 (524–540 q acre^−1^) had the highest yield and Punjab Ratta and PTNI-25 had the lowest. Marketable yield had a greater genotypic control (*R*_A_ = 209.19 and *R*_B_ = 173.05), and PTNI-203, PTNI-8, and PTNI-67 yielded 459–485 q acre^−1^. The marketable yield of 501 to 328 q acre^−1^ (35% reduction) at 6 dS m^−1^ is shown in **Table 3**.

### Root growth and biomass

Root biomass was strongly influenced by genotype (*R*_A_ = 32.15) and then on salinity (*R*_B_ = 18.23). The tolerance genotypes PTNI-11, PTNI-25, and PTNI-20 had the maximum root biomass (72–74 g) and Punjab Ratta and PTNI-19 had less. The weight of roots of field-grown plants was higher than that of pot-grown plants (74.8 vs. 51.7 g). Root dry matter was also the same (*R*_A_ = 16.41 and *R*_B_ = 11.01), but Punjab Chhuhara, PTNI-25, and PTNI-203 at the same time retained a higher dry weight, as highlighted in **Table 3**.

### Alterations in physiological and biochemical traits of different lines under salinity stress

**Figure 3:**
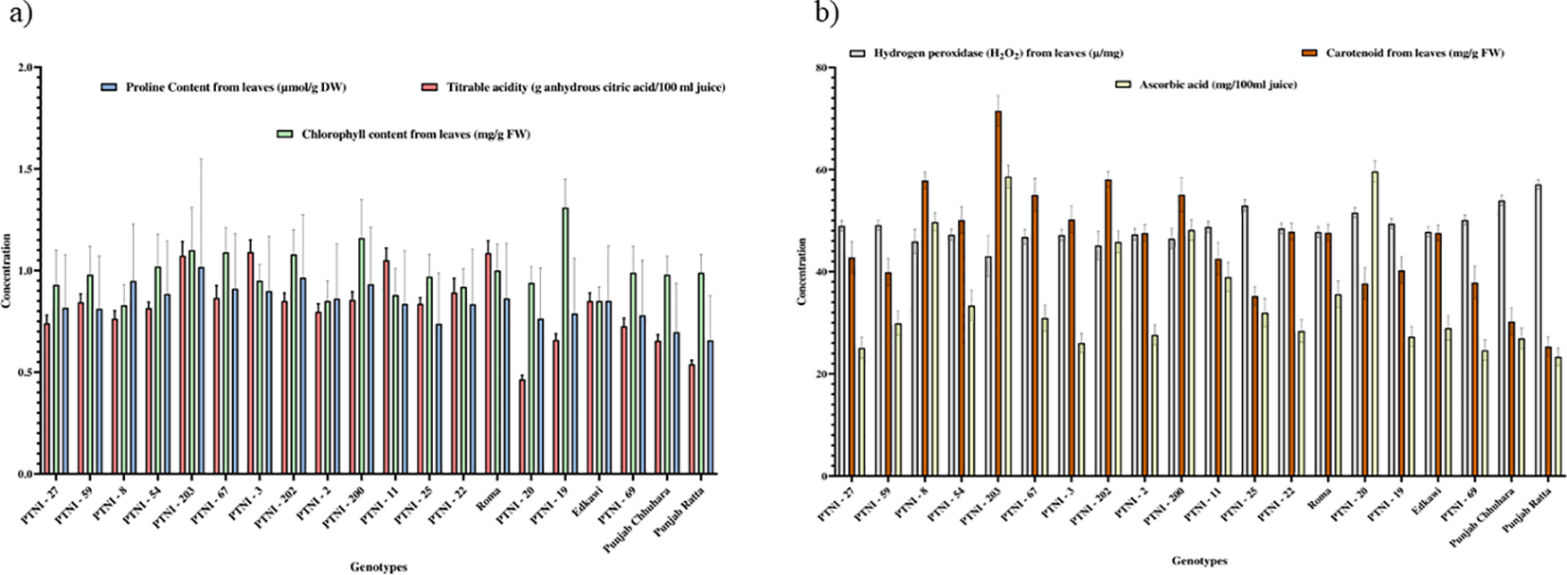
Comparative analysis of primary metabolites and antioxidant capacity among tomato genotypes under salinity stress. **(a)** Primary metabolites. **(b)** Antioxidant-related traits.

**Figure 4:**
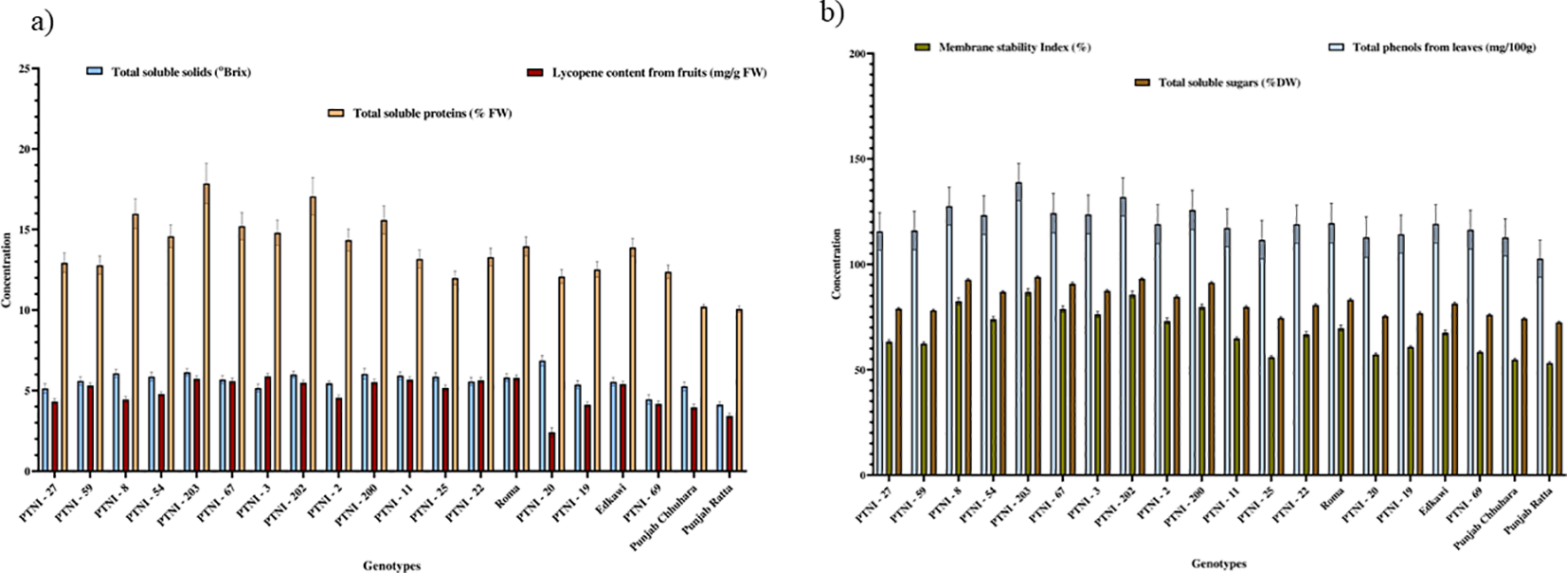
Illustration of fruit quality, nutritional attributes and membrane stability traits in tomato genotypes under salinity stress. **(a)** Fruit quality and nutritional traits. **(b)** Membrane stability and related physiological traits.

Salinity significantly altered physiological and biochemical traits in tomato genotypes (**Figures 3**, **4**). TA declined by 50% at higher saline conditions, as shown in **Figure 3a**. PTNI-203, PTNI-3, and Roma genotypes were statistically at par with each other and recorded the highest TA (1.07 to 1.09). However, Ratta showed the lowest TA (0.54) and PTNI-20 (0.46). Proline accumulation drastically increased to 1.02 from 0.66 µmol g^−1^ DW (50% increase) from susceptible to resistant lines. Genotypes PTNI-203 and PTNI-202 accumulated 1.02 and 0.97 µmol g^−1^ DW, respectively. However, the susceptible check Punjab Ratta showed a significant lower proline accumulation of 0.66 µmol g^−1^ DW. At higher salinity stress (6 ds m^−1^), chlorophyll content declined from 1.37 to 0.87 mg/g FW, showing accelerated pigment degradation and impaired chloroplast stability with increasing stress duration. Nevertheless, PTNI-202 and PTNI-67 retained pigment levels exceeding 1.33 mg/g FW, which contributed to enhanced photosynthetic stability under stress.

Higher TSS was observed in PTNI-20 (6.86 °Brix), followed by PTNI-203 (6.14 °Brix) and PTNI-8 (6.07 °Brix). The lowest values were recorded in Punjab Ratta (4.15 °Brix). The degree of soluble protein content was raised to 15.56% in salt stress conditions. Protein accumulation was greatest in PTNI-203 (17.86%) and PTNI-202 (17.06%), which indicated that structural and enzymatic defense was via active proteome remodeling (**Figure 3b**). The range of lycopene was 3.42 mg/g FW (Punjab Ratta) to 5.89 mg/g FW (PTNI-3) and statistically at par with PTNI-203.

Oxidative stress indicators varied significantly among genotypes, with H_2_O_2_ levels higher in susceptible genotypes, as shown in **Figure 4a**. Punjab Ratta, Punjab Chhuhara and PTNI-25 exhibited significantly higher H_2_O_2_ levels, whereas PTNI-203 and PTNI-202 recorded significantly lower H_2_O_2_ levels. Carotenoid levels also decreased in a similar fashion, although PTNI-203 and PTNI-8 showed an exceptionally high level (71.51 and 57.88 mg g^−1^ FW) compared to Punjab Ratta (25.36 mg g^−1^ FW). The highest concentration of ascorbic acid in PTNI-203 and PTNI-20 showed more oxidative buffering capacity, and the lowest values were recorded in Punjab Ratta and PTNI-69 with a range of 23–25 mg 100 mL^−1^.

The stability index of the membrane (MSI) was also found to decrease to 53.17% under salinity conditions in susceptible lines (**Figure 4b**). That is a sign of ionic-imbalance stress, but PTNI-203 and PTNI-202 retained a better stability index than Punjab Chhuhara. Total soluble sugars increased 72.45% DW (control) to 93.95% DW in saline conditions, and tolerant lines PTNI-203, PTNI-202, and PTNI-8 showed higher than 92% DW. However, in resistant lines, PTNI-203 and PTNI-202 exhibited a significantly higher phenol content of 139.99 and 131.94 mg 100 g^−1^ FW, respectively.

### Principal component analysis for trait changes and genotypes in salt stress

**Figure 5:**
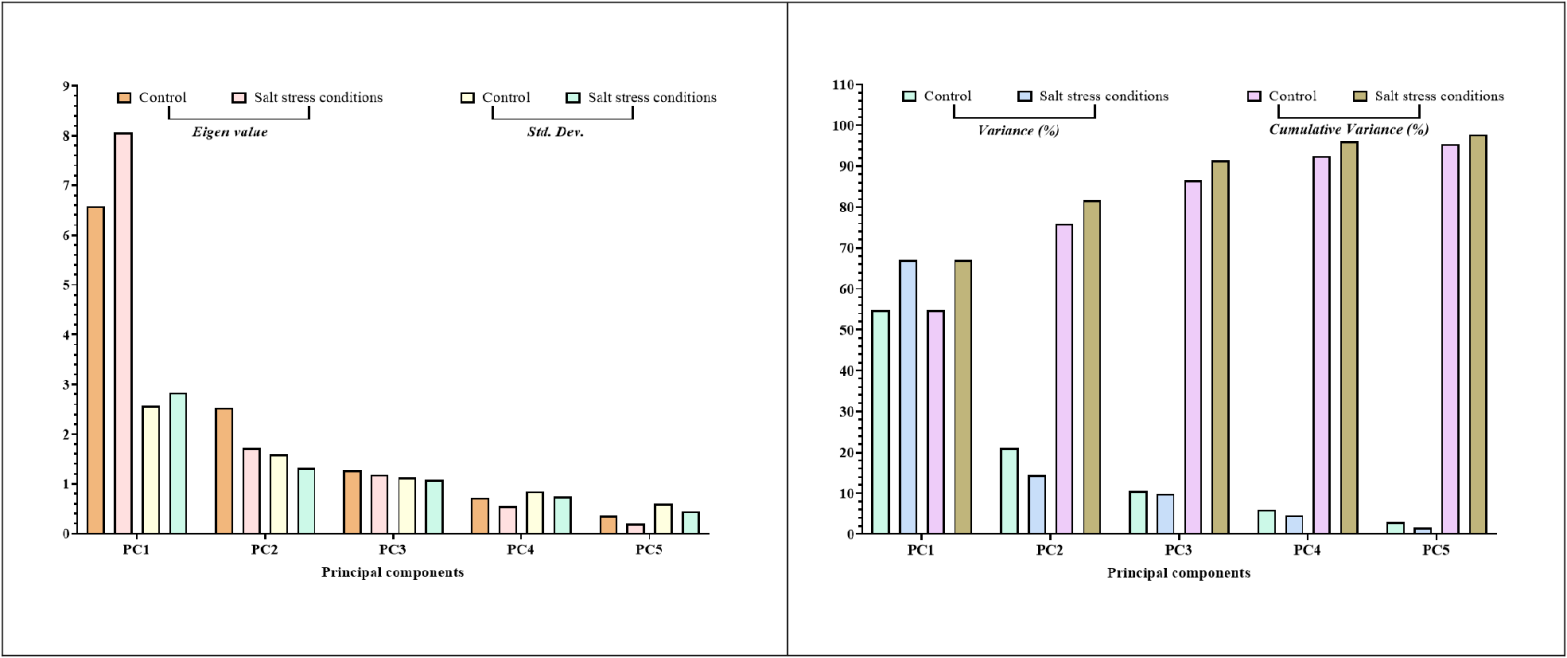
Eigenvalues, standard deviations, and percentage variance explained by principal components derived from morpho-physiological and yield traits of tomato genotypes under control and salinity stress conditions.

**Figure 5** summarizes the principal component analysis (PCA) results for tomato traits under control and salt stress conditions. Under salinity stress, the first two principal components (PC1 and PC2) explained 81.69% of the total variance (67.16% and 14.53%, respectively), indicating that most trait variation was captured within these two axes. Subsequent components contribute progressively less to total variance in both conditions.

Distinct patterns of trait variation were observed between control and salinity conditions (**Supplementary Figure 1**). Under salinity stress, PC1 was primarily associated with growth and yield-related traits, whereas PC2 was mainly associated with phenological and reproductive traits. A clear separation of genotypes into tolerant and susceptible groups was observed under salinity stress, indicating differential trait responses and adaptation mechanisms. In contrast, the control indicated a slightly wider distribution of the variance in Dim1 and Dim2 and a smaller level of differentiation between genotypes, therefore capturing baseline variance in the absence of salt stress. **Supplementary Tables 1**-**8** provide concise information on tomato trait and genotype coordinates on principal component axes (Dim1–Dim5), correlation coefficients, squared cosine (cos^2^), and contribution percentages showing each trait’s relative influence, thus enabling thorough understanding of morpho-physiological and yield traits for genotype performance across varied environments.

### Correlation matrices demonstrate strong growth coupling and augmented trade-offs in salinity

**Figure 6:**
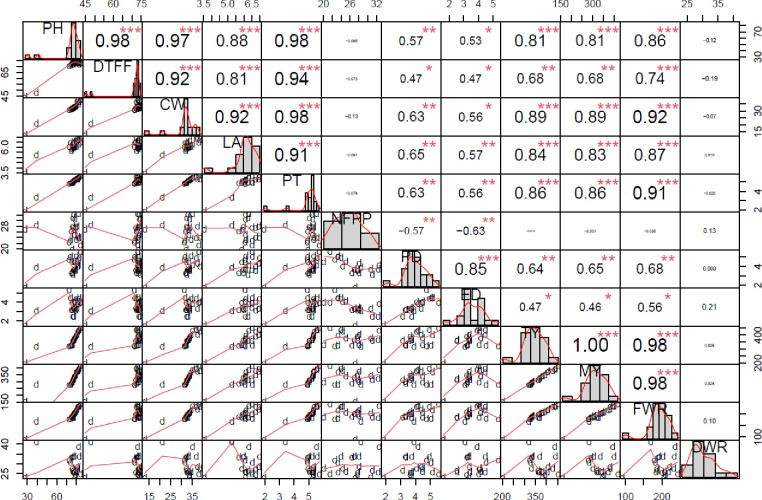
Pairwise correlation matrices for key tomato growth and yield traits under salt stress conditions. Each cell displays the Pearson correlation coefficient between two traits. Statistical significance is denoted by asterisks: *P < 0.05, **P < 0.01, and ***P < 0.001, providing a measure of confidence for each correlation. Trait abbreviations are as follows: PH stands for plant height; DTFF is days to 50% flowering; CW is canopy width; LA is leaf area; PT represents pericarp thickness; FWR denotes fruit weight of roots; DWR is dry weight of roots; ED refers to equatorial diameter; PD is polar diameter; TY is total yield; MY is marketable yield; and NFPP is number of fruits per plant.

As shown in **Figure 6**, all vegetative growth characteristics of tomatoes including plant height, canopy width, and the area of leaves are strongly correlated (*R* = 0.88 and above) and the biomass development of tomato has a high level of synchrony irrespective of the environmental conditions. Flowering days are closely correlated with both growth characteristics in each case dependent on the vigor of the plant. The predictors of yield also exhibit strong positive relationships with growth characteristics during stress, whereas under salt stress, trade-offs increase: the NFPP is negatively correlated with fruit size and vegetative traits, which means that there is a resource-allocation change, likely to decrease the size of fruits in high-fruiting genotypes. Notably, the total and marketable yield is almost perfectly correlated (*r* = 0.99–100) to support fruit conversion efficiency under saline conditions. These results indicate that salinity stress strengthens the dependence of yield on vegetative growth traits while introducing trade-offs between fruit number and size.

### Heatmap profiling finds genotype-specific combinations of performance and traits of tolerance and yield traits

**Figure 7:**
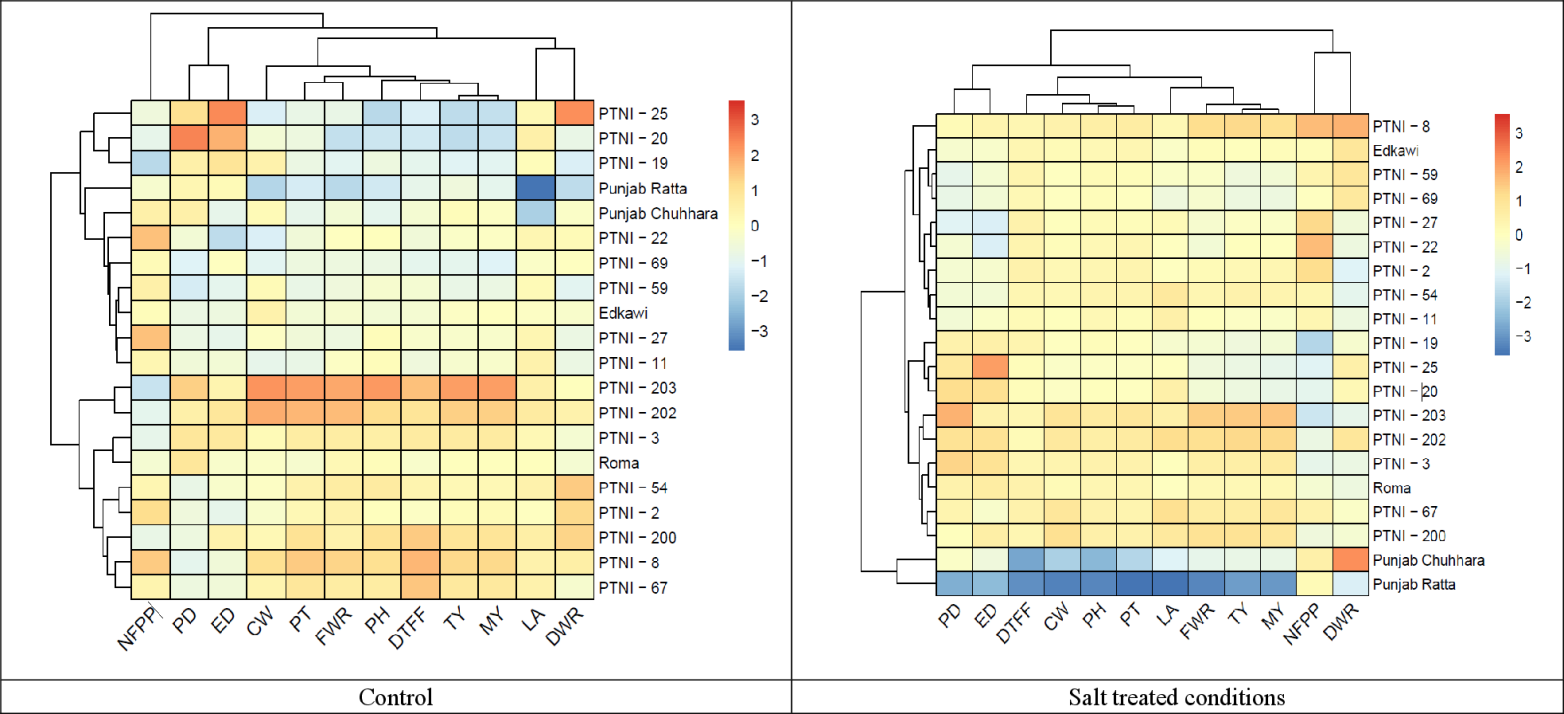
Heatmap showing standardized genotype-by-trait profiles of tomato genotypes under control and salt stress conditions. The heatmap illustrates the relative performance of genotypes across multiple morpho-physiological and yield traits. Color intensity represents relative trait expression, where warmer colors (yellow to red) indicate higher values and cooler colors (blue) represent lower values. The clustering pattern highlights genotype-specific responses and the stability of certain genotypes under salinity stress.

As shown in **Figure 7**, heatmap analysis revealed clear genotype × trait patterns under both control and salinity conditions. Under control conditions, high-performing genotypes like PTNI-203, PTNI-200, and PTNI-8 show higher values on the growth and yield traits and low-performance genotypes like Punjab Ratta show consistently lower values on the traits. It has been observed that genotypes such as PTNI-8 and PTNI-203 exhibit greater stability under salt stress conditions, in terms of standardized values of relevant traits, and in general, they are more resilient, with many of them showing decline under salt stress. The grouping of every heatmap identifies clusters of genotypes and traits that covary in performance with yield-related parameters identifying parallel tolerance in a selection of lines with stress markers displaying distinct variation.

### Trait integration and genetic control through factor analysis

**Figure 8:**
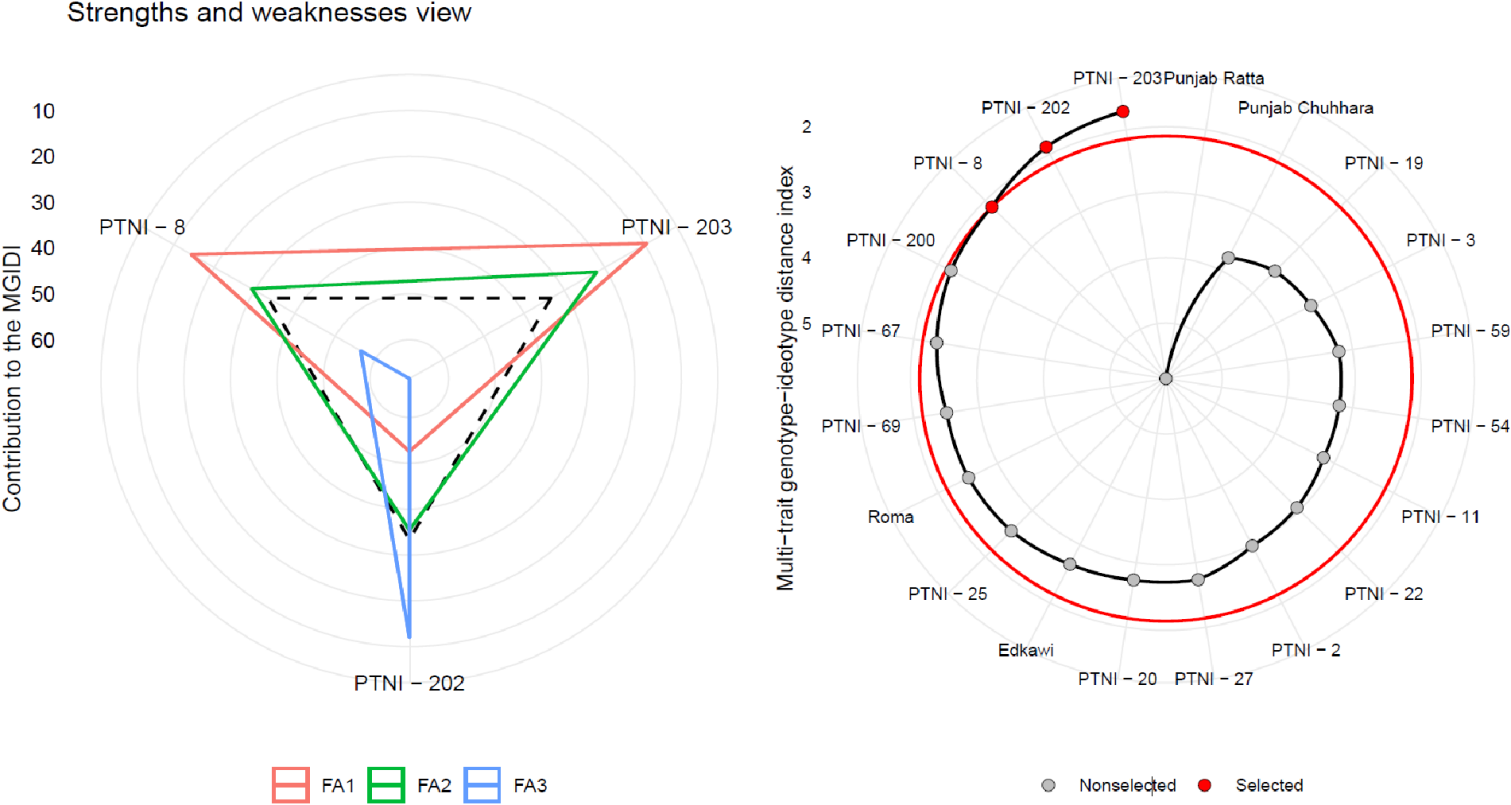
Identification of elite tomato genotypes combining salt tolerance and multi-trait superiority using the Multi-Trait Genotype–Ideotype Distance Index (MGIDI) and strengths–weaknesses analysis. The MGIDI biplot illustrates the relative distance of each genotype from the ideotype (ideal genotype), where shorter distances indicate superior performance across multiple traits. The strengths–weaknesses view highlights the contribution of different factor axes (FA1–FA3) representing vegetative growth, yield components, and root traits, respectively, enabling the identification of genotypes with balanced trait performance under salinity stress.

Multivariate genotype ideotype distance index (MGIDI)-based analysis and factor integration provided a comprehensive assessment of salt tolerance across tomato genotypes (**Table 4**, **Figure 8**). The radar chart showed the relative contribution of the three major factor axes (FA_1_, FA_2_, and FA_3_) to the MGIDI in which low contributions imply closeness to the ideotype and the relative high performance. Among the analyzed genotypes, PTNI-203, PTNI-202, and PTNI-8 showed the lowest values in terms of MGIDI, which denotes balanced traits expression and enhanced salt tolerance, and the susceptible checks Punjab Chhuhara and Punjab Ratta were located further away in the ideotype, which implies a lack of adaptability to salinity. The MGIDI biplot further confirmed that these tolerant genotypes were clustered near the ideotype, which further affirmed their choice as the most promising tolerant lines to salinity stress.

**Table 4:** Trait contributions to latent factors explaining salt tolerance in tomato genotypes.

Traits	FA1	FA2	FA3	Communality	Uniqueness
Plant height	0.94	−0.17	−0.06	0.91	0.09
Days to 50% flowering	−0.88	0.07	0.19	0.82	0.18
Canopy width	0.97	−0.16	−0.04	0.96	0.04
Leaf area	0.91	−0.24	−0.17	0.92	0.08
Pericarp thickness	0.97	−0.14	0.01	0.96	0.04
Number of fruits per plant	0.02	0.90	−0.19	0.85	0.15
Polar diameter	0.22	−0.87	−0.03	0.80	0.20
Equatorial diameter	0.13	−0.93	−0.17	0.91	0.09
Total yield	0.93	0.06	0.01	0.87	0.13
Marketable yield	0.97	−0.02	−0.05	0.94	0.06
Fresh weight of roots	0.17	−0.24	−0.83	0.78	0.22
Dry weight of roots	0.01	0.23	−0.86	0.80	0.20

FA_1_, FA_2_, and FA_3_ represent factor axes derived from factor analysis used for calculating the Multi-Trait Genotype–Ideotype Distance Index (MGIDI). Communality represents the proportion of variance explained by the extracted factors, whereas uniqueness indicates the unexplained variance associated with each trait.

Factor analysis subdivided the assessed characteristics into three main components that were related to vegetative growth (FA_1_), yield (FA_2_), and root characteristics (FA_3_) (**Table 5**). The vegetative characteristics had high positive loadings with low uniqueness (0.04 to 0.09) and positive selection responses to selection (*h*^2^ = 0.712 to 0.994), which were consistent with strong genetic control. The reproductive characteristics, days to 50% flowering, the NFPP, and the size of the fruit had slight decreases (−0.6% to −5.46%), and the total and marketable yield showed an improvement, with the marketable yield showing a significant selection response of 16.3%. Root traits showed high negative loadings (–0.83 to –0.86) but positive selection response, indicating their independent contribution to stress tolerance. The results indicate that vegetative growth and root development are predominantly enhanced, reproductive traits are mildly constrained, while yield stability demonstrates the integrative resilience of the tomato genotypes.

**Table 5:** Selection differential, selection gain, and heritability of key tomato traits grouped by factor axes (FA_1_–FA_3_) under saline stress.

Traits	Factor	X_o_	X_s_	SD	SD%	h²	SG	SG%	Direction
Plant height	FA1	88.50	89.70	1.28	1.45	0.98	1.26	1.43	↑
Days to 50% flowering	FA1	49.10	48.70	−0.38	−0.77	0.81	−0.31	−0.63	↓
Canopy width	FA1	39.20	39.90	0.75	1.92	0.98	0.74	1.89	↑
Leaf area	FA1	7.22	7.54	0.32	4.44	0.94	0.30	4.17	↑
Pericarp thickness	FA1	5.59	5.62	0.04	0.65	0.99	0.04	0.64	↑
Number of fruits per plant	FA1	467.00	460.00	−6.75	−1.45	0.99	−6.71	−1.44	↓
Polar diameter	FA1	420.00	417.00	−2.53	−0.60	1.00	−2.51	−0.60	↓
Equatorial diameter	FA2	28.90	27.30	−1.58	−5.46	1.00	−1.57	−5.43	↓
Total yield	FA2	4.70	4.73	0.03	0.74	0.97	0.03	0.72	↑
Marketable yield	FA2	4.23	4.93	0.70	16.50	0.99	0.69	16.30	↑
Fresh weight of roots	FA3	59.50	66.60	7.12	12.00	0.71	5.06	8.52	↑
Dry weight of roots	FA3	27.80	29.10	1.24	4.46	0.85	1.06	3.81	↑

Selection differential (SD), selection gain (SG), and broad-sense heritability (*h*²) estimates of key morpho-physiological and yield traits of tomato genotypes under salinity stress. FA_1_, FA_2_, and FA_3_ represent factor axes derived from factor analysis. X_o_ represents the mean of the original population and X_s_ represents the mean of the selected population. SD and SG indicate selection differential and predicted genetic gain, respectively.

## Discussion

Salinity (applied through irrigation water at different EC levels, expressed as dS m^-1^) significantly affected tomato growth, yield, and physiological processes, with genotype-specific variation in tolerance. The high performance of genotypes including PTNI-203, PTNI-202, and PTNI-8 under salinity stress (6 dS m^−1^) reflects their ability to maintain growth and reproductive activity under osmotic and ionic stress conditions. They are predicted to have an increased osmotic adjustment and retention of turgor due to their increased plant height, canopy width, and leaf area, which allows them to continue cell division and photosynthetic capacity despite salt buildup (Ludwiczak et al., 2021). On the other hand, sensitive genotypes, i.e., Punjab Ratta and Punjab Chhuhara, showed significant losses in vegetative and yield parameters due to association with ionic intoxication and inhibited water uptake (Zafar et al., 2022; Balasubramaniam et al., 2023).

Salinity stress delayed flowering across genotypes, likely due to disruption of carbohydrate metabolism and hormonal balance (Abbas et al., 2010; Cho et al., 2017). However, tolerant lines had comparatively fixed flowering periods, implying an increased control of osmotic stress reaction and antioxidative metabolism. The maintenance of fruit morphological characters, especially pericarp thickness, in resistant lines is also an indication of structural adaptation, which is most likely facilitated by enhanced calcium transportation and cell wall quality, hence supporting fruit firmness and prolonged shelf life in saline environments (Fernández-Garcí et al., 2004; Jia et al., 2023).

Yield traits were strongly influenced by genotype, indicating genetic regulation of salinity tolerance. The positive relationship between vegetative vigor, fruit set, and total yield observed under saline stress has also been reported to be linked to previous reports that have claimed that photosynthetic activity remains resilient under salinity to support assimilate translocation to reproductive sinks (Feller et al., 2017; Wang et al., 2018). These results indicate coordinated regulation of growth and yield under stress. Root characteristics were established to be very important in salinity resilience. It was found that tolerant genotypes had greater root fresh and dry biomass, which reflected the ability to compartmentalize ions and osmotic adjustment ability (Llanes et al., 2021; Kou et al., 2022). These results are consistent with the observed relationship between growth, root development, and yield stability under salinity stress. This supports their ability to sustain water uptake under ionic stress (Munns and Tester, 2008).

Multivariate analyses including PCA, correlation matrices, and MGIDI provided supporting data for multi-trait tolerance (Olivoto et al., 2022; Debnath et al., 2024). PCA was able to form a distinction between tolerant and susceptible genotypes, with the first two dimensions explaining over 80% of the total variance when saline was used. These strong correlations between yield and growth characteristics and trade-offs in fruit number and size are indicative of a differentiation in resource use across salt stress (Maggio et al., 2004; Alam et al., 2021; Oliveira et al., 2022). The MGIDI and the strengths weaknesses analyses revealed that PTNI-203, PTNI-202, and PTNI-8 were the closest approximations of the ideotype and combined high yield, vegetative vigor, and root biomass with only a few drawbacks among traits. These findings highlight the fact that salt tolerance in tomato is a multi-genic, integrative process, which is controlled through the co-occurring preservation of growth, root performance, and yield efficiency (Guo et al., 2022). The identified genotypes, which are reported to have superior MGIDI performance, have a positive content that can be used in breeding programs in the pursuit to improve productivity under salt-affected conditions.

Biochemical responses further supported genotypic tolerance (Liu et al., 2023). These proline profiles in tolerant lines have demonstrated a consistent production of osmolytes, which assists in safeguarding enzymes and neutralizing reactive oxygen species (Zulfiqar and Ashraf, 2023). These high chlorophyll levels in resistant lines confirm the presence of a cohesive osmotic network that connects sugar, proline, and membrane stability, thereby supporting cellular functions when exposed to salinity.

Higher TSS in resistant lines is an addition to flavor density and process yield (Mizrahi et al., 1988; Sgherri et al., 2008; Jin et al., 2023). Genotypic differences in biochemical traits were evident under stress (Fan et al., 2024). This was may be due to the induction of the protective proteins (dehydrins and HSPs). The salinity has a strong modulatory effect on the antioxidant and pigment metabolism whereby significant genotype differentiation is evident among tomato accessions (Timperio et al., 2008). The gradual procession of increasing lycopene shows an induction of carotenoids to moderate oxidative stress and an adaptive metabolic response (Del Giudice et al., 2017; Massaretto et al., 2018).

Oxidative stress indicators such as H_2_O_2_ were higher in sensitive genotypes, whereas tolerant genotypes maintained lower levels. This reflects efficient antioxidant regulation (Mittova et al., 2003). This balanced activation will prevent the overuse of substrates by ROS, hence preserving redox activity (Aon et al., 2010). The preservation of carotenoids is a stressful condition of salinity, which indicates the increased activity of photoprotective quenching and ROS neutralization, which is in line with the antioxidative interaction between xanthophylls and ascorbate (Duarte et al., 2013). In general, tolerant genotypes maintained pigment integrity and antioxidant potential, and those sensitive lines displayed quick pigment degradation, a characteristic of photochemical destabilisation in the presence of ions. The current resistance of ascorbate to tolerant lines is consistent with the reported results of Hossain and Dietz (2016), who observed improved ascorbate turnover as a key characteristic of redox resilience in saline environments.

Membrane stability was higher in tolerant genotypes, indicating improved cellular protection mechanisms (Rodríguez-Rosales et al., 1999). Higher phenolic content further supports enhanced antioxidant capacity in tolerant genotypes (Frary et al., 2010; Agius et al., 2022). The concentration of phenols in tolerant genotypes suggests a biochemical redistribution to enhance radical quenching and wall-tightening abilities, which are essential qualities of redox adaptation in response to stress conditions. Phenolics create lignin–protein complexes and stabilize membranes of tolerant lines (Rao and Zheng, 2025).

## Conclusion

Salinity significantly reduced vegetative growth, reproductive performance, and yield of tomato; however, the extent of reduction was strongly genotype-dependent. Genotypes PTNI-203, PTNI-202, PTNI-8, and PTNI-200 consistently maintained superior horticultural traits under salinity stress (6 dS m^−1^), suggesting high adaptive capabilities of the genotype to osmotic adjustment, ionic regulation, and stress-tolerance processes. These genotypes also provided better marketable yield as well as the fruit morphology compared to salt-sensitive varieties like Punjab Ratta and Punjab Chhuhara. Multivariate analysis (PCA and MGIDI) confirmed that these genotypes were the closest to the ideotype, exhibiting stable multi-trait performance across both field and pot conditions. The results highlight the importance of identifying salt-tolerant tomato genotypes to support productivity in salt-affected areas and to offer potential solutions to cultivation and breeding projects to improve tomato production.

## Data Availability

The original contributions presented in the study are included in the article/**Supplementary Material**. Further inquiries can be directed to the corresponding authors.

## Supplementary material

Supplementary Figure 1 Principal component analysis biplots illustrating genotype and trait distributions under control and salt stress conditions.

Supplementary Table 1 Coordinates of tomato traits on principal component axes under control and salt stress conditions.

Supplementary Table 2 Correlation of traits with principal components (Dim1–Dim5). Values close to ±1 indicate a strong association of the trait with the respective principal component

Supplementary Table 3 Squared cosine (cos²) values of morpho-physiological and yield traits on principal components (Dim1–Dim 5), indicating the quality of representation of each trait on the respective component.

Supplementary Table 4 Contribution (%) of morpho-physiological and yield traits to principal components (Dim1–Dim 5) indicating the relative importance of each trait in defining the components.

Supplementary Table 5 Coordinates of tomato genotypes in the PCA space (Dim1–Dim 5) based on morpho-physiological and yield traits.

Supplementary Table 6 PCA cos^2^ of 20 tomato genotypes across principal components (Dim1–Dim 5).

Supplementary Table 7 Contribution of tomato genotypes to PCA dimensions (Dim1–Dim 5).

Supplementary Table 8 Principal component analysis (PCA) loadings of 12 tomato growth, yield, and fruit traits and proportion of variance explained by each component.
