# Supplementary material for: Genotypic resilience and fruit quality responses of tomato (Solanum lycopersicum L.) in progressive salinity stress across diverse cultivation conditions
Source: Front Plant Sci. 2026 Jun 16;17:1786599. doi: 10.3389/fpls.2026.1786599 (PMC13314448; doi:10.3389/fpls.2026.1786599)
Supplement: Supplementary Figure 1 — Principal component analysis biplots illustrating genotype and trait distributions under control and salt stress conditions. [file SupplementaryFile1.docx]

| 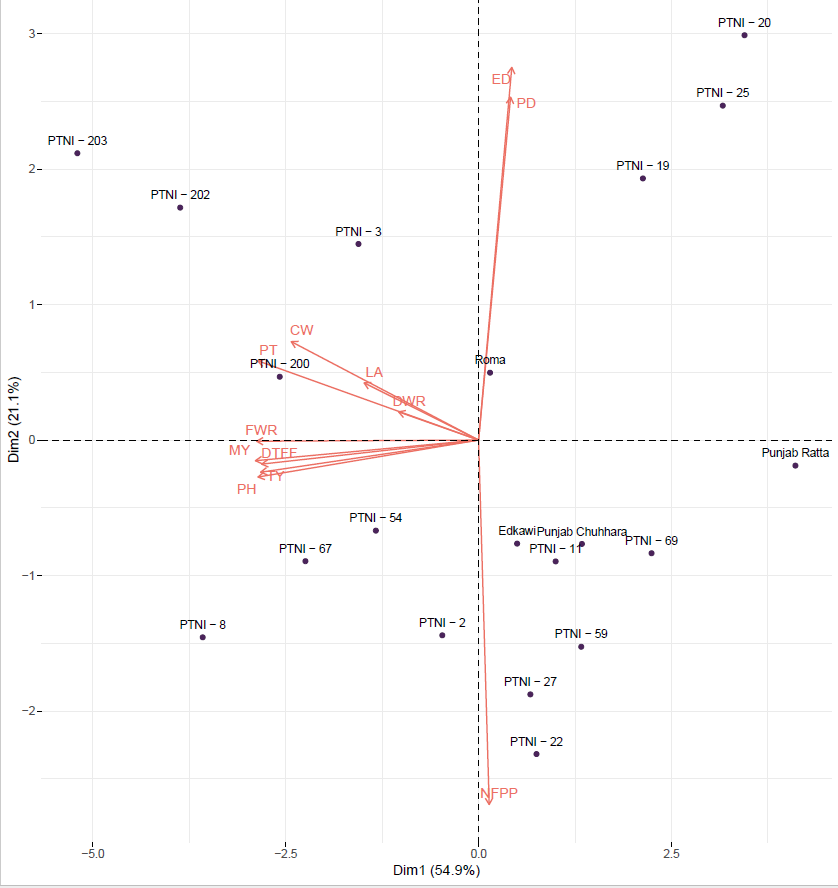 | 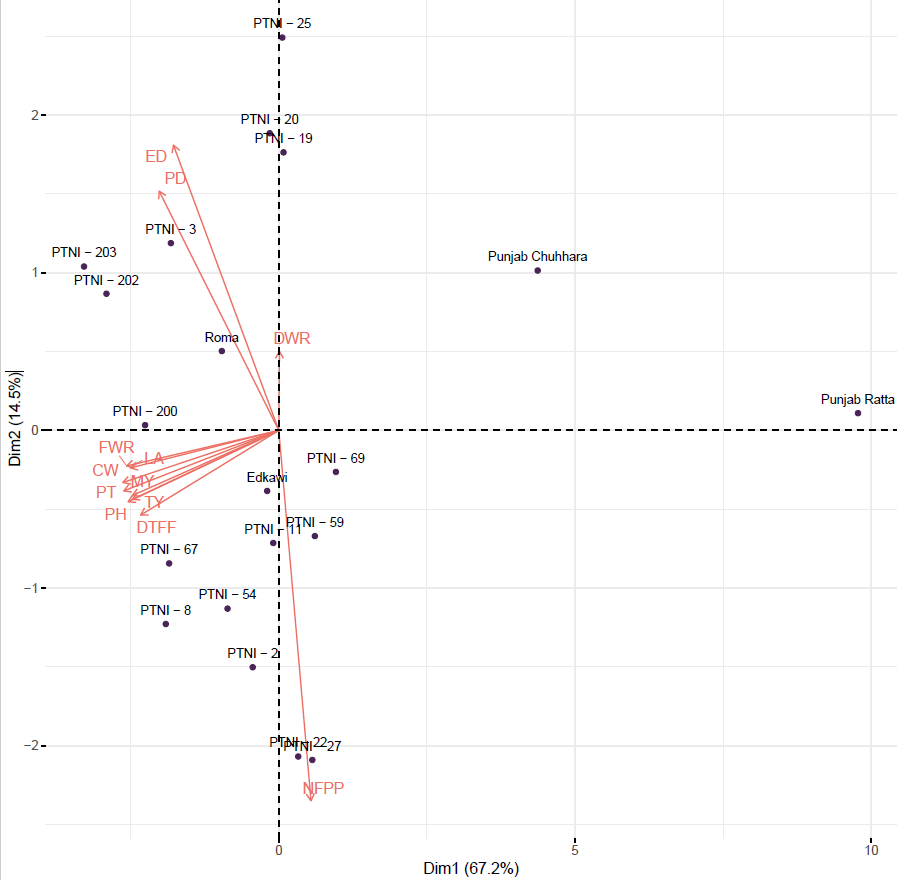 |
| --- | --- |
| Control | Salt treated conditions |

**Supplementary Figure 1.** Principal component analysis biplots illustrating genotype and trait distributions under control and salt stress conditions

**Supplementary Table 1.** Coordinates of tomato traits on principal component axes under control and salt stress conditions.

| **Trait** | **Control** | | | | | **Salt stress conditions** | | | | |
| --- | --- | --- | --- | --- | --- | --- | --- | --- | --- | --- |
|  | **Dim 1** | **Dim2** | **Dim 3** | **Dim 4** | **Dim 5** | **Dim 1** | **Dim2** | **Dim 3** | **Dim 4** | **Dim 5** |
| PH | -0.960 | -0.092 | 0.064 | -0.110 | -0.020 | -0.946 | -0.168 | -0.158 | -0.211 | -0.046 |
| DTFF | -0.944 | -0.060 | 0.088 | 0.114 | -0.130 | -0.867 | -0.200 | -0.261 | -0.361 | -0.028 |
| CW | -0.815 | 0.244 | 0.204 | -0.250 | -0.016 | -0.979 | -0.123 | -0.076 | -0.071 | -0.077 |
| LA | -0.498 | 0.142 | -0.579 | -0.614 | 0.091 | -0.931 | -0.088 | 0.032 | -0.059 | 0.105 |
| PT | -0.961 | 0.196 | -0.053 | 0.055 | -0.021 | -0.973 | -0.143 | -0.040 | -0.145 | -0.005 |
| NFPP | 0.046 | -0.903 | -0.204 | 0.049 | 0.288 | 0.202 | -0.873 | 0.332 | -0.050 | 0.281 |
| PD | 0.139 | 0.849 | 0.184 | 0.101 | 0.459 | -0.751 | 0.564 | 0.058 | 0.161 | 0.214 |
| ED | 0.144 | 0.923 | -0.219 | 0.089 | -0.151 | -0.661 | 0.672 | 0.078 | -0.168 | 0.167 |
| TY | -0.948 | -0.080 | 0.234 | 0.176 | 0.032 | -0.919 | -0.163 | 0.151 | 0.315 | -0.028 |
| MY | -0.971 | -0.051 | 0.173 | 0.112 | 0.064 | -0.918 | -0.153 | 0.147 | 0.322 | -0.054 |
| FWR | -0.965 | -0.003 | -0.096 | 0.062 | 0.096 | -0.955 | -0.084 | 0.170 | 0.180 | -0.095 |
| DWR | -0.348 | 0.070 | -0.815 | 0.427 | 0.000 | 0.004 | 0.186 | 0.939 | -0.257 | -0.122 |

**Supplementary Table 2.** Correlation of traits with principal components (Dim1–Dim5). Values close to ±1 indicate a strong association of the trait with the respective

| **Trait** | **Control** | | | | | **Salt stress conditions** | | | | |
| --- | --- | --- | --- | --- | --- | --- | --- | --- | --- | --- |
|  | **Dim 1** | **Dim2** | **Dim 3** | **Dim 4** | **Dim 5** | **Dim 1** | **Dim2** | **Dim 3** | **Dim 4** | **Dim 5** |
| PH | -0.960 | -0.092 | 0.064 | -0.110 | -0.020 | -0.946 | -0.168 | -0.158 | -0.211 | -0.046 |
| DTFF | -0.944 | -0.060 | 0.088 | 0.114 | -0.130 | -0.867 | -0.200 | -0.261 | -0.361 | -0.028 |
| CW | -0.815 | 0.244 | 0.204 | -0.250 | -0.016 | -0.979 | -0.123 | -0.076 | -0.071 | -0.077 |
| LA | -0.498 | 0.142 | -0.579 | -0.614 | 0.091 | -0.931 | -0.088 | 0.032 | -0.059 | 0.105 |
| PT | -0.961 | 0.196 | -0.053 | 0.055 | -0.021 | -0.973 | -0.142 | -0.040 | -0.145 | -0.005 |
| NFPP | 0.046 | -0.903 | -0.204 | 0.049 | 0.288 | 0.202 | -0.873 | 0.332 | -0.050 | 0.281 |
| PD | 0.139 | 0.849 | 0.184 | 0.101 | 0.459 | -0.751 | 0.564 | 0.058 | 0.161 | 0.214 |
| ED | 0.144 | 0.923 | -0.219 | 0.089 | -0.151 | -0.661 | 0.672 | 0.078 | -0.168 | 0.167 |
| TY | -0.948 | -0.080 | 0.234 | 0.176 | 0.032 | -0.919 | -0.163 | 0.151 | 0.315 | -0.028 |
| MY | -0.971 | -0.051 | 0.173 | 0.112 | 0.064 | -0.918 | -0.153 | 0.147 | 0.322 | -0.054 |
| FWR | -0.965 | -0.003 | -0.096 | 0.062 | 0.096 | -0.955 | -0.084 | 0.170 | 0.180 | -0.094 |
| DWR | -0.348 | 0.070 | -0.815 | 0.427 | 0.000 | 0.004 | 0.186 | 0.939 | -0.257 | -0.122 |

**Supplementary Table 3.** Squared cosine (cos²) values of morpho-physiological and yield traits on principal components (Dim1–Dim 5), indicating the quality of representation of each trait on the respective component.

| **Trait** | **Control** | | | | | **Salt stress conditions** | | | | |
| --- | --- | --- | --- | --- | --- | --- | --- | --- | --- | --- |
|  | **Dim 1** | **Dim2** | **Dim 3** | **Dim 4** | **Dim 5** | **Dim 1** | **Dim2** | **Dim 3** | **Dim 4** | **Dim 5** |
| PH | 0.921 | 0.008 | 0.004 | 0.012 | 0.000 | 0.894 | 0.028 | 0.025 | 0.045 | 0.002 |
| DTFF | 0.890 | 0.004 | 0.008 | 0.013 | 0.017 | 0.752 | 0.040 | 0.068 | 0.130 | 0.001 |
| CW | 0.664 | 0.059 | 0.042 | 0.062 | 0.000 | 0.958 | 0.015 | 0.006 | 0.005 | 0.006 |
| LA | 0.248 | 0.020 | 0.335 | 0.377 | 0.008 | 0.867 | 0.008 | 0.001 | 0.003 | 0.011 |
| PT | 0.923 | 0.039 | 0.003 | 0.003 | 0.000 | 0.947 | 0.020 | 0.002 | 0.021 | 0.000 |
| NFPP | 0.002 | 0.815 | 0.042 | 0.002 | 0.083 | 0.041 | 0.762 | 0.110 | 0.003 | 0.079 |
| PD | 0.019 | 0.721 | 0.034 | 0.010 | 0.211 | 0.563 | 0.318 | 0.003 | 0.026 | 0.046 |
| ED | 0.021 | 0.852 | 0.048 | 0.008 | 0.023 | 0.437 | 0.452 | 0.006 | 0.028 | 0.028 |
| TY | 0.899 | 0.006 | 0.055 | 0.031 | 0.001 | 0.844 | 0.026 | 0.023 | 0.099 | 0.001 |
| MY | 0.943 | 0.003 | 0.030 | 0.012 | 0.004 | 0.843 | 0.023 | 0.022 | 0.104 | 0.003 |
| FWR | 0.932 | 0.000 | 0.009 | 0.004 | 0.009 | 0.913 | 0.007 | 0.029 | 0.033 | 0.009 |
| DWR | 0.121 | 0.005 | 0.664 | 0.182 | 0.000 | 0.005 | 0.035 | 0.881 | 0.066 | 0.015 |

**Supplementary Table 4.** Contribution (%) of morpho-physiological and yield traits to principal components (Dim1–Dim 5) indicating the relative importance of each trait in defining the components.

| **Trait** | **Control** | | | | | **Salt stress conditions** | | | | |
| --- | --- | --- | --- | --- | --- | --- | --- | --- | --- | --- |
|  | **Dim 1** | **Dim2** | **Dim 3** | **Dim 4** | **Dim 5** | **Dim 1** | **Dim2** | **Dim 3** | **Dim 4** | **Dim 5** |
| PH | 13.99 | 0.33 | 0.32 | 1.68 | 0.11 | 11.10 | 1.63 | 2.12 | 7.92 | 1.04 |
| DTFF | 13.52 | 0.14 | 0.61 | 1.81 | 4.76 | 9.33 | 2.30 | 5.81 | 23.12 | 0.39 |
| CW | 10.09 | 2.35 | 3.27 | 8.69 | 0.07 | 11.89 | 0.87 | 0.49 | 0.90 | 2.93 |
| LA | 3.77 | 0.79 | 26.33 | 52.54 | 2.34 | 10.75 | 0.45 | 0.09 | 0.62 | 5.51 |
| PT | 14.01 | 1.52 | 0.22 | 0.43 | 0.12 | 11.74 | 1.17 | 0.14 | 3.75 | 0.01 |
| NFPP | 0.03 | 32.19 | 3.27 | 0.33 | 23.16 | 0.51 | 43.92 | 9.37 | 0.45 | 39.49 |
| PD | 0.29 | 28.49 | 2.65 | 1.43 | 59.08 | 6.99 | 18.33 | 0.28 | 4.61 | 22.90 |
| ED | 0.32 | 33.65 | 3.78 | 1.10 | 6.36 | 5.42 | 26.05 | 0.52 | 5.03 | 14.01 |
| TY | 13.66 | 0.25 | 4.30 | 4.30 | 0.28 | 10.48 | 1.52 | 1.93 | 17.59 | 0.40 |
| MY | 14.32 | 0.10 | 2.34 | 1.74 | 1.14 | 10.47 | 1.35 | 1.83 | 18.46 | 1.44 |
| FWR | 14.16 | 0.00 | 0.72 | 0.53 | 2.57 | 11.32 | 0.40 | 2.45 | 5.78 | 4.46 |
| DWR | 1.84 | 0.19 | 52.19 | 25.44 | 0.00 | 0.00 | 2.00 | 74.97 | 11.77 | 7.42 |

**Supplementary Table 5.** Coordinates of tomato genotypes in the PCA space (Dim1–Dim 5) based on morpho-physiological and yield traits.

| **Genotypes** | **Control** | | | | | **Salt stress conditions** | | | | |
| --- | --- | --- | --- | --- | --- | --- | --- | --- | --- | --- |
|  | **Dim 1** | **Dim2** | **Dim 3** | **Dim 4** | **Dim 5** | **Dim 1** | **Dim2** | **Dim 3** | **Dim 4** | **Dim 5** |
| PTNI - 27 | 0.671 | -1.877 | 0.075 | -0.885 | 0.411 | 0.566 | -2.089 | -0.454 | -0.380 | 0.033 |
| PTNI - 59 | 1.330 | -1.525 | 0.290 | -1.317 | -0.582 | 0.608 | -0.670 | 0.513 | -1.116 | -0.494 |
| PTNI - 8 | -3.577 | -1.456 | -0.316 | 0.263 | 0.080 | -1.908 | -1.228 | 2.424 | -0.047 | 0.215 |
| PTNI - 54 | -1.332 | -0.668 | -1.359 | 0.488 | -0.306 | -0.866 | -1.130 | -0.757 | 0.273 | -0.027 |
| PTNI - 203 | -5.201 | 2.116 | 1.249 | 0.062 | 0.414 | -3.288 | 1.039 | -0.712 | 1.597 | -0.337 |
| PTNI - 67 | -2.245 | -0.894 | 0.318 | -0.236 | -0.287 | -1.852 | -0.843 | 0.071 | 0.394 | 0.136 |
| PTNI - 3 | -1.557 | 1.447 | 0.592 | 0.073 | 0.091 | -1.822 | 1.187 | -0.673 | 0.677 | 0.228 |
| PTNI - 202 | -3.869 | 1.715 | 0.105 | -0.041 | -0.067 | -2.910 | 0.867 | 0.964 | 0.478 | -0.222 |
| PTNI - 2 | -0.470 | -1.441 | -1.072 | 0.680 | 0.433 | -0.441 | -1.502 | -0.808 | 0.199 | 0.561 |
| PTNI - 200 | -2.578 | 0.467 | -0.857 | 0.561 | -1.106 | -2.257 | 0.033 | -0.264 | 0.528 | -0.535 |
| PTNI - 11 | 0.999 | -0.896 | -0.001 | -0.826 | 0.058 | -0.094 | -0.714 | -0.635 | -0.171 | 0.193 |
| PTNI - 25 | 3.165 | 2.468 | -2.871 | 1.110 | 0.008 | 0.059 | 2.491 | -0.001 | -1.223 | 0.605 |
| PTNI - 22 | 0.750 | -2.317 | -0.649 | -0.055 | 1.018 | 0.328 | -2.068 | -0.417 | -0.136 | 0.604 |
| Roma | 0.149 | 0.497 | 0.451 | -0.156 | 0.689 | -0.962 | 0.503 | -0.691 | 0.109 | 0.210 |
| PTNI - 20 | 3.447 | 2.987 | -0.137 | -0.936 | 0.936 | -0.154 | 1.884 | -0.225 | -0.856 | 0.703 |
| PTNI - 19 | 2.130 | 1.931 | 0.788 | -1.311 | -0.761 | 0.079 | 1.763 | -1.117 | -0.513 | -0.559 |
| Edkawi | 0.499 | -0.764 | 0.247 | -0.437 | -0.314 | -0.197 | -0.384 | 0.667 | -0.512 | -0.603 |
| PTNI - 69 | 2.242 | -0.835 | -0.697 | -0.190 | -0.809 | 0.964 | -0.263 | 0.330 | -1.063 | -0.780 |
| Punjab Chuhhara | 1.339 | -0.767 | 1.300 | 1.249 | 0.670 | 4.370 | 1.014 | 2.930 | 0.977 | 0.232 |
| Punjab Ratta | 4.107 | -0.189 | 2.544 | 1.904 | -0.576 | 9.777 | 0.109 | -1.145 | 0.787 | -0.166 |

**Supplementary Table 6.** PCA cos^2^ of 20 tomato genotypes across principal components (Dim1–Dim 5)

| **Genotypes** | **Control** | | | | | **Salt stress conditions** | | | | |
| --- | --- | --- | --- | --- | --- | --- | --- | --- | --- | --- |
|  | **Dim 1** | **Dim2** | **Dim 3** | **Dim 4** | **Dim 5** | **Dim 1** | **Dim2** | **Dim 3** | **Dim 4** | **Dim 5** |
| PTNI - 27 | 0.082 | 0.644 | 0.001 | 0.143 | 0.031 | 0.062 | 0.850 | 0.040 | 0.028 | 0.000 |
| PTNI - 59 | 0.272 | 0.358 | 0.013 | 0.267 | 0.052 | 0.143 | 0.174 | 0.102 | 0.481 | 0.094 |
| PTNI - 8 | 0.793 | 0.131 | 0.006 | 0.004 | 0.000 | 0.314 | 0.130 | 0.507 | 0.000 | 0.004 |
| PTNI - 54 | 0.385 | 0.097 | 0.401 | 0.052 | 0.020 | 0.243 | 0.413 | 0.185 | 0.024 | 0.000 |
| PTNI - 203 | 0.807 | 0.134 | 0.047 | 0.000 | 0.005 | 0.705 | 0.070 | 0.033 | 0.166 | 0.007 |
| PTNI - 67 | 0.747 | 0.118 | 0.015 | 0.008 | 0.012 | 0.726 | 0.150 | 0.001 | 0.033 | 0.004 |
| PTNI - 3 | 0.441 | 0.381 | 0.064 | 0.001 | 0.001 | 0.552 | 0.234 | 0.075 | 0.076 | 0.009 |
| PTNI - 202 | 0.813 | 0.160 | 0.001 | 0.000 | 0.000 | 0.798 | 0.071 | 0.087 | 0.022 | 0.005 |
| PTNI - 2 | 0.051 | 0.481 | 0.266 | 0.107 | 0.043 | 0.052 | 0.606 | 0.176 | 0.011 | 0.085 |
| PTNI - 200 | 0.685 | 0.022 | 0.076 | 0.032 | 0.126 | 0.805 | 0.000 | 0.011 | 0.044 | 0.045 |
| PTNI - 11 | 0.294 | 0.236 | 0.000 | 0.201 | 0.001 | 0.007 | 0.398 | 0.314 | 0.023 | 0.029 |
| PTNI - 25 | 0.390 | 0.237 | 0.321 | 0.048 | 0.000 | 0.000 | 0.748 | 0.000 | 0.181 | 0.044 |
| PTNI - 22 | 0.071 | 0.678 | 0.053 | 0.000 | 0.131 | 0.020 | 0.802 | 0.033 | 0.003 | 0.068 |
| Roma | 0.015 | 0.169 | 0.139 | 0.017 | 0.325 | 0.488 | 0.133 | 0.252 | 0.006 | 0.023 |
| PTNI - 20 | 0.514 | 0.386 | 0.001 | 0.038 | 0.038 | 0.005 | 0.679 | 0.010 | 0.140 | 0.094 |
| PTNI - 19 | 0.397 | 0.326 | 0.054 | 0.150 | 0.051 | 0.001 | 0.616 | 0.247 | 0.052 | 0.062 |
| Edkawi | 0.127 | 0.298 | 0.031 | 0.097 | 0.050 | 0.029 | 0.110 | 0.332 | 0.195 | 0.271 |
| PTNI - 69 | 0.703 | 0.098 | 0.068 | 0.005 | 0.091 | 0.318 | 0.024 | 0.037 | 0.387 | 0.208 |
| Punjab Chuhhara | 0.245 | 0.080 | 0.231 | 0.213 | 0.061 | 0.639 | 0.034 | 0.287 | 0.032 | 0.002 |
| Punjab Ratta | 0.609 | 0.001 | 0.234 | 0.131 | 0.012 | 0.979 | 0.000 | 0.013 | 0.006 | 0.000 |

**Supplementary Table 7.** Contribution of tomato genotypes to PCA dimensions (Dim1–Dim 5)

| **Genotypes** | **Control** | | | | | **Salt stress conditions** | | | | |
| --- | --- | --- | --- | --- | --- | --- | --- | --- | --- | --- |
|  | **Dim 1** | **Dim2** | **Dim 3** | **Dim 4** | **Dim 5** | **Dim 1** | **Dim2** | **Dim 3** | **Dim 4** | **Dim 5** |
| PTNI - 27 | 0.34 | 6.96 | 0.02 | 5.46 | 2.37 | 0.20 | 12.58 | 0.87 | 1.28 | 0.03 |
| PTNI - 59 | 1.34 | 4.60 | 0.33 | 12.11 | 4.74 | 0.23 | 1.29 | 1.12 | 11.06 | 6.10 |
| PTNI - 8 | 9.72 | 4.19 | 0.39 | 0.48 | 0.09 | 2.26 | 4.34 | 24.98 | 0.02 | 1.16 |
| PTNI - 54 | 1.35 | 0.88 | 7.27 | 1.66 | 1.31 | 0.46 | 3.68 | 2.44 | 0.66 | 0.02 |
| PTNI - 203 | 20.54 | 8.85 | 6.13 | 0.03 | 2.40 | 6.71 | 3.11 | 2.16 | 22.65 | 2.84 |
| PTNI - 67 | 3.83 | 1.58 | 0.40 | 0.39 | 1.15 | 2.13 | 2.05 | 0.02 | 1.38 | 0.46 |
| PTNI - 3 | 1.84 | 4.14 | 1.38 | 0.04 | 0.12 | 2.06 | 4.06 | 1.93 | 4.07 | 1.30 |
| PTNI - 202 | 11.37 | 5.81 | 0.04 | 0.01 | 0.06 | 5.25 | 2.16 | 3.95 | 2.03 | 1.24 |
| PTNI - 2 | 0.17 | 4.10 | 4.52 | 3.23 | 2.63 | 0.12 | 6.50 | 2.78 | 0.35 | 7.88 |
| PTNI - 200 | 5.05 | 0.43 | 2.89 | 2.19 | 17.13 | 3.16 | 0.00 | 0.30 | 2.47 | 7.16 |
| PTNI - 11 | 0.76 | 1.58 | 0.00 | 4.76 | 0.05 | 0.01 | 1.47 | 1.71 | 0.26 | 0.93 |
| PTNI - 25 | 7.61 | 12.03 | 32.42 | 8.60 | 0.00 | 0.00 | 17.88 | 0.00 | 13.29 | 9.16 |
| PTNI - 22 | 0.43 | 10.61 | 1.65 | 0.02 | 14.51 | 0.07 | 12.32 | 0.74 | 0.16 | 9.12 |
| Roma | 0.02 | 0.49 | 0.80 | 0.17 | 6.65 | 0.57 | 0.73 | 2.03 | 0.10 | 1.10 |
| PTNI - 20 | 9.03 | 17.63 | 0.07 | 6.12 | 12.25 | 0.01 | 10.23 | 0.21 | 6.51 | 12.36 |
| PTNI - 19 | 3.44 | 7.36 | 2.44 | 11.99 | 8.10 | 0.00 | 8.96 | 5.31 | 2.34 | 7.81 |
| Edkawi | 0.19 | 1.15 | 0.24 | 1.33 | 1.38 | 0.02 | 0.42 | 1.89 | 2.33 | 9.09 |
| PTNI - 69 | 3.82 | 1.38 | 1.91 | 0.25 | 9.15 | 0.58 | 0.20 | 0.46 | 10.04 | 15.20 |
| Punjab Chuhhara | 1.36 | 1.16 | 6.64 | 10.88 | 6.28 | 11.85 | 2.96 | 36.52 | 8.47 | 1.35 |
| Punjab Ratta | 12.81 | 0.07 | 25.44 | 25.29 | 4.64 | 59.31 | 0.03 | 5.58 | 5.50 | 0.69 |

**Supplementary Table 8.** Principal component analysis (PCA) loadings of 12 tomato growth, yield, and fruit traits and proportion of variance explained by each component.

| **Genotypes** | **Control** | | | | | **Salt stress conditions** | | | | |
| --- | --- | --- | --- | --- | --- | --- | --- | --- | --- | --- |
|  | **Dim 1** | **Dim2** | **Dim 3** | **Dim 4** | **Dim 5** | **Dim 1** | **Dim2** | **Dim 3** | **Dim 4** | **Dim 5** |
| PH | -0.374 | -0.058 | 0.056 | -0.130 | -0.034 | -0.333 | -0.128 | -0.145 | -0.281 | -0.102 |
| DTFF | -0.368 | -0.037 | 0.078 | 0.134 | -0.218 | -0.305 | -0.152 | -0.241 | -0.481 | -0.062 |
| CW | -0.318 | 0.153 | 0.181 | -0.295 | -0.026 | -0.345 | -0.093 | -0.070 | -0.095 | -0.171 |
| LA | -0.194 | 0.089 | -0.513 | -0.725 | 0.153 | -0.328 | -0.067 | 0.029 | -0.078 | 0.235 |
| PT | -0.374 | 0.123 | -0.047 | 0.065 | -0.035 | -0.343 | -0.108 | -0.037 | -0.194 | -0.012 |
| NFPP | 0.018 | -0.567 | -0.181 | 0.058 | 0.481 | 0.071 | -0.663 | 0.306 | -0.067 | 0.628 |
| PD | 0.054 | 0.534 | 0.163 | 0.120 | 0.769 | -0.264 | 0.428 | 0.053 | 0.215 | 0.479 |
| ED | 0.056 | 0.580 | -0.194 | 0.105 | -0.252 | -0.233 | 0.510 | 0.072 | -0.224 | 0.374 |
| TY | -0.370 | -0.050 | 0.207 | 0.207 | 0.053 | -0.324 | -0.123 | 0.139 | 0.419 | -0.063 |
| MY | -0.378 | -0.032 | 0.153 | 0.132 | 0.107 | -0.324 | -0.116 | 0.135 | 0.430 | -0.120 |
| FWR | -0.376 | -0.002 | -0.085 | 0.073 | 0.160 | -0.337 | -0.064 | 0.156 | 0.240 | -0.211 |
| DWR | -0.136 | 0.044 | -0.722 | 0.504 | 0.001 | 0.001 | 0.141 | 0.866 | -0.343 | -0.272 |
